# PupilEXT: Flexible Open-Source Platform for High-Resolution Pupillometry in Vision Research

**DOI:** 10.3389/fnins.2021.676220

**Published:** 2021-06-18

**Authors:** Babak Zandi, Moritz Lode, Alexander Herzog, Georgios Sakas, Tran Quoc Khanh

**Affiliations:** ^1^Laboratory of Lighting Technology, Department of Electrical Engineering and Information Technology, Technical University of Darmstadt, Darmstadt, Germany; ^2^Interactive Graphic Systems, Department of Computer Science, Technical University of Darmstadt, Darmstadt, Germany

**Keywords:** pupillometry, pupil measurement, stereo camera, vision research, pupil diameter, eye tracking, open source

## Abstract

The human pupil behavior has gained increased attention due to the discovery of the intrinsically photosensitive retinal ganglion cells and the afferent pupil control path’s role as a biomarker for cognitive processes. Diameter changes in the range of 10^–2^ mm are of interest, requiring reliable and characterized measurement equipment to accurately detect neurocognitive effects on the pupil. Mostly commercial solutions are used as measurement devices in pupillometry which is associated with high investments. Moreover, commercial systems rely on closed software, restricting conclusions about the used pupil-tracking algorithms. Here, we developed an open-source pupillometry platform consisting of hardware and software competitive with high-end commercial stereo eye-tracking systems. Our goal was to make a professional remote pupil measurement pipeline for laboratory conditions accessible for everyone. This work’s core outcome is an integrated cross-platform (macOS, Windows and Linux) pupillometry software called PupilEXT, featuring a user-friendly graphical interface covering the relevant requirements of professional pupil response research. We offer a selection of six state-of-the-art open-source pupil detection algorithms (Starburst, Swirski, ExCuSe, ElSe, PuRe and PuReST) to perform the pupil measurement. A developed 120-fps pupillometry demo system was able to achieve a calibration accuracy of 0.003 mm and an averaged temporal pupil measurement detection accuracy of 0.0059 mm in stereo mode. The PupilEXT software has extended features in pupil detection, measurement validation, image acquisition, data acquisition, offline pupil measurement, camera calibration, stereo vision, data visualization and system independence, all combined in a single open-source interface, available at https://github.com/openPupil/Open-PupilEXT.

## Introduction

The pupil diameter is an essential metric in visual neuroscience, as it has a direct impact on the retinal irradiance, visual acuity and visual performance of the eye (Campbell, 1957; Campbell and Gubisch, 1966; Woodhouse, 1975; Schwiegerling, 2000). Since the early days of pupillary research (Reeves, 1918), the modeling of the pupil light response and its retinal processing path was the main focus of investigations (Zandi and Khanh, 2021). Additionally, the pupil diameter is used as a biomarker in research disciplines such as cognitive science (Aminihajibashi et al., 2020; Cherng et al., 2020; Clewett et al., 2020; Sibley et al., 2020), circadian photoentrainment (Münch et al., 2012; Bonmati-Carrion et al., 2016; Spitschan et al., 2019; Tähkämö et al., 2019; Van Egroo et al., 2019), clinical diagnostics (Lim et al., 2016; Joyce et al., 2018; Chougule et al., 2019) or neuroscience (Schwalm and Jubal, 2017; Carle et al., 2019). Pupil changes of 0.015 to 0.5 mm are the range of interest in such studies, leading to increased resolution and robustness requirements for pupil measurement equipment. Closed commercial eye-tracking systems are common in pupil examinations, associated with high investments without offering the possibilities of validating the pupil detection’s measurement accuracy. Additionally, with closed systems, it is not possible to identify the applied pupil detection algorithm, making it challenging to reproduce experiments since small inaccuracies in a range of 0.01 mm could propagate errors to the statistical evaluation of the pupil diameter. Apart from commercial solutions, there is currently a lack of an end-to-end open-source measurement platform that can be easily set up for high-precision pupillometry under laboratory conditions. Therefore, we developed a freely available hardware and software platform for pupil measurements to support the increased interest of interdisciplinary research groups in studying the pupil behavior. Our proposed platform is a comprehensive solution for performing accurate, verifiable and reproducible pupil examinations, competitive with high-end commercial stereo eye-tracking systems.

**FIGURE 1:**
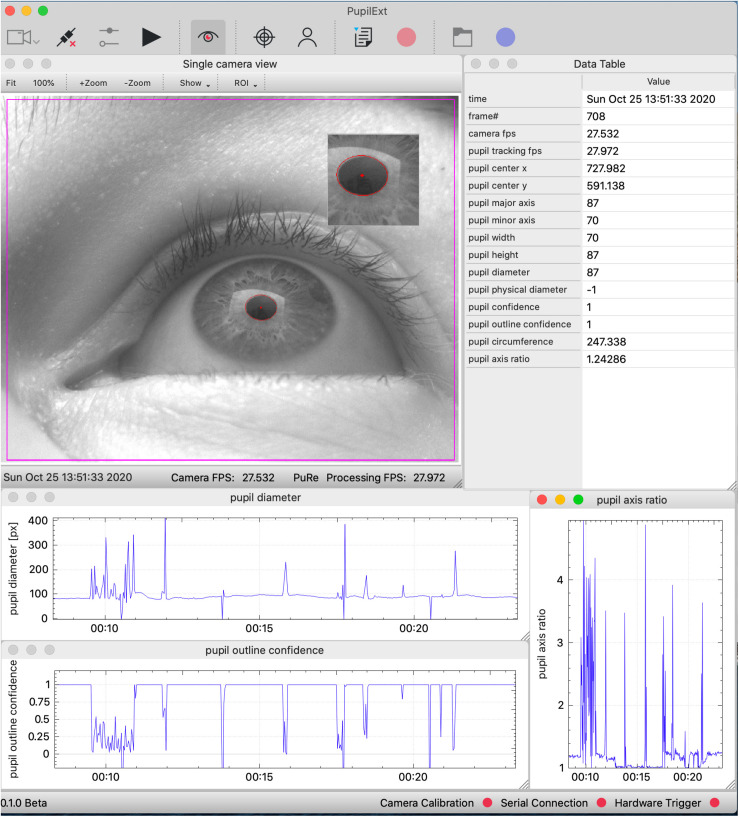
The graphical user interface of the *PupilEXT* software during a pupil measurement with one connected industrial camera. The measured pupil values are listed in real-time in a table or can be visualized graphically. We provide a selection of six state-of-the-art pupil detection algorithms from the literature. Stereo camera systems can be connected and calibrated seamlessly to acquire the absolute pupil diameter. The accuracy of a pupil measurement or calibration can be verified by implemented routines.

The core outcome of this work is an integrated cross-platform (macOS, Windows and Linux) pupillometry software called *PupilEXT*, featuring a user-friendly graphical interface (C++, QT), covering the relevant requirements of professional pupil behavior research (Figure 1). The open-source philosophy offers insight into how the pupil measurement framework performs, motivating to more transparency in collecting pupil data. We aimed to provide a plug-and-play integrated hardware and software platform, allowing interdisciplinary research groups a precise pupil behavior research without high investments. The proposed software is designed to incorporate high-resolution industrial cameras that can be run either individually or in a stereo camera arrangement. We guarantee a stable frame rate and synchronous operation of stereo cameras by using a microcontroller as an external hardware trigger. The integrated solution with hardware and software is provided in a way that even scientists with a non-technical background can reproduce the system. Users simply need to purchase industrial cameras and run the proposed *PupilEXT* software.

Inspired by the eye-tracking software *EyeRecToo* (Santini et al., 2017) from Santini et al., we offer end-users a selection of six state-of-the-art open-source pupil detection algorithms (*Starburst*, *Swirski*, *ExCuSe*, *Else*, *PuRe* and *PuReST*) to perform the pupil measurement. The system allows researchers to report the used pupil algorithm with the respective parameters since the pupil detection method itself could influence the captured data. Additionally, end-users will be able to determine the pupil diameter from externally acquired image sequences through the software suite. The integrated platform is available to other research groups as an open-source project, ensuring continuous development in the future. We aimed to bridge the gap between visual neuroscience or experimental psychology and engineering sciences, making professional remote pupil measurements under laboratory conditions accessible for everyone, without suffering the features of commercial solutions.

The first section of this work deals with the scientific background of pupil behavior research and the rising popularity of this topic, from which we derive the motivation of the proposed pupil measurement platform. Based on that, the current state of pupillometry and the availability of suitable open-source frameworks are highlighted. Next, we conducted a meta-analysis of existing pupil detection algorithms from the literature intending to select and integrate appropriate algorithms in the proposed *PupilEXT* software. The functionality of the platform is covered by starting with the hardware components, consisting of cameras, microcontroller and a near-infrared (NIR) illumination. Here, we describe the possible hardware topologies with which end-users can conduct a pupil measurement or offline analysis of external captured images. In particular, we show the possibilities of validating a pupil measurement and camera calibration with the *PupilEXT* software. Finally, the performance of the system is demonstrated with an experiment concerning the pupil light response, clarifying the provided pupil metrics for reliable data evaluation.

## The Rising Popularity of Pupil Light Response Research

The human retina contains receptors with distinct photopigments, capable of transforming light quanta of different wavelengths λ into frequency-coded action potentials with information on color and brightness features from a visual stimulus. Photoreceptors in the retina are classified according to their broad spectral sensitivity in the visible spectrum range and respective peak response λ_Peak_. In the photopic adapted eye, the retinal image-forming pathway is mainly controlled by the short-wavelength (S, λ_Peak_ 420 nm), medium-wavelength (M, λ_Peak_ 535 nm) and long-wavelength (L, λ_Peak_ 565 nm) sensitive cones (Stockman and Sharpe, 2000; Solomon and Lennie, 2007; Lucas et al., 2014). At scotopic and mesopic light conditions, the more sensitive rods (λ_Peak_ 498 nm) dominate the vision. Both cones and rods transmit, depending on the adaptation state of the eye, integrated signals in different stages through ganglion cells to the visual cortex of the brain (Van Meeteren, 1978; Smith et al., 2008; Jennings and Martinovic, 2014). In 1924, the International Commission on Illumination (CIE) introduced the photopic luminous efficiency function *V*(λ) to estimate the visual effectiveness of light spectra for humans (Bodmann, 1992; Sharpe et al., 2005; Sagawa, 2006).

A standard value in estimating the human brightness perception is the luminance *L* given in cd/m^2^, which is a *V*(λ) weighted photometric quantity (Berman et al., 1990; Lennie et al., 1993; Withouck et al., 2013). The luminance is merely a first approximation of the brightness perception, as only the additive contribution of L- and M-cones to the image-forming pathway is managed by *V*(λ) (CIE, 2011; Besenecker and Bullough, 2017; Hermans et al., 2018; Zandi et al., 2021). Since 1926, about eight pupil models were proposed that integrated the luminance as a main dependent parameter, assuming that the afferent pupil control pathway can be described by a *V*(λ) weighted quantity (Holladay, 1926; Crawford, 1936; Moon and Spencer, 1944; de Groot and Gebhard, 1952; Stanley and Davies, 1995; Blackie and Howland, 1999; Barten, 1999; Watson and Yellott, 2012; Zandi et al., 2020).

The discovery of a new type of receptors in the outer retina called intrinsically photosensitive retinal ganglion cells (ipRGCs) was a turning point of vision science (Provencio et al., 1998, 2000; Gooley et al., 2001; Berson et al., 2002; Hattar, 2002; Mure, 2021), which has led to a rethinking of classical retinal processing models. This subset of ganglion cells are part of the non-image-forming mechanism of the eye because of their projection to regions of the suprachiasmatic nucleus (SCN) and olivary pretectal nucleus (OPN) (Ruby et al., 2002; Berson, 2003; Hattar et al., 2003; Do et al., 2009; Ecker et al., 2010; Allen et al., 2019; Do, 2019). As a result, the ipRGCs can modulate the circadian rhythm (Freedman, 1999; Brainard et al., 2001; Thapan et al., 2001; Rea and Figueiro, 2018; Truong et al., 2020) and pupil light response (Lucas et al., 2001, 2020; Gamlin et al., 2007; Young and Kimura, 2008; Barrionuevo et al., 2018; Murray et al., 2018) via a processing path that works independently of the classical image-forming pathway (Hattar et al., 2006; Güler et al., 2008; Schmidt et al., 2014; Spitschan, 2019a). Recent studies showed that the pupil light response cannot be described by the *V*(λ) weighted luminance alone, making a revision of classical pupil models necessary (Zandi et al., 2018, 2020; Spitschan, 2019b; Zele et al., 2019). Therefore, one key topic in pupillary research is the development of a valid empirical model (Zandi et al., 2020), providing a spectral and time-variant function with dynamic receptor weighting to predict the temporal aperture across individuals (Rao et al., 2017; Zandi and Khanh, 2021). When using stimulus spectra along the Planckian locus for triggering the pupil light response, it is essential in measurements that amplitudes in the range of 0.1 to 0.4 mm are captured accurately to specify intrasubject variability (Kobashi et al., 2012) in a pupil model. However, a special requirement for pupil measurements arises when the pupil is used as a biomarker for quantifying the cognitive state (Morad et al., 2000; Merritt et al., 2004; Murphy et al., 2014; Ostrin et al., 2017; Tkacz-Domb and Yeshurun, 2018; Hu et al., 2019; Van Egroo et al., 2019; de Winter et al., 2021; Van der Stoep et al., 2021) or clinical symptoms of diseases (Hreidarsson, 1982; Maclean and Dhillon, 1993; Connelly et al., 2014; Lim et al., 2016; Granholm et al., 2017; Wildemeersch et al., 2018; Chougule et al., 2019). Cognitive processes such as memory load, arousal, circadian status, or sleepiness have a transient impact (Watson and Yellott, 2012) on the pupil diameter with aperture changes of 0.015 to 0.53 mm (Beatty and Wagoner, 1978; Beatty, 1982; Schluroff et al., 1986; Jepma and Nieuwenhuis, 2011; Pedrotti et al., 2014; Bombeke et al., 2016; Tsukahara et al., 2016; Winn et al., 2018), making the reproducibility of such effects difficult if the accuracy of the measurement equipment has not been sufficiently validated.

**FIGURE 2:**
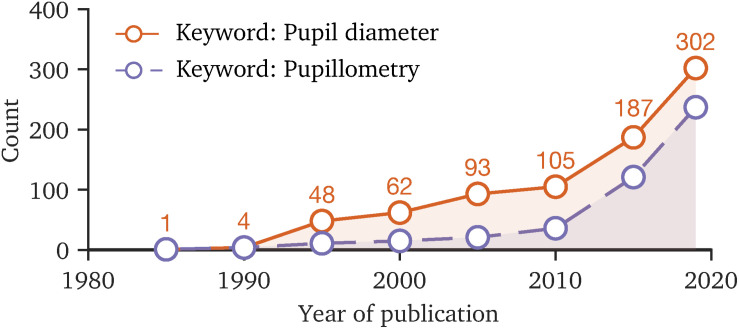
The number of publications with the keywords “pupil diameter” and “pupillometry” since 1985 to 2019, based on the Web of Science database. The rising count of publications in recent years indicates that the topic of pupil behavior is becoming more important. Due to the interdisciplinary field of research, standardization of measurement methodology and data processing is favorable, making study results comparable.

Today, the pupil behavior has become an interdisciplinary field of research (La Morgia et al., 2018; Schneider et al., 2020; Joshi, 2021; Pinheiro and da Costa, 2021) in which the number of involved scientists rises, as the trend of the number of publications with the keywords “pupil diameter” or “pupillometry” reveals (Figure 2). The renewed attention to the temporal pupil aperture (Binda and Gamlin, 2017), its application in clinical diagnostics (Granholm et al., 2017; Joyce et al., 2018; Chougule et al., 2019; Kercher et al., 2020; Tabashum et al., 2021) and increasing popularity of chromatic pupillometry (Rukmini et al., 2017; Crippa et al., 2018) topics requires additional efforts in terms of standardization and provision of consistent tools, contributing to comparability in measurement and pre-processing methodologies. For instance, one key point of standardization is the prevention of artificially induced changes to raw data by the used tools, as in cognitive or vision-related pupillary research small diameter margins are of interest. The main methodology factors that could influence the research results or reliability of pupil behavior studies are as follows:

(1) Number and depth of described experimental metrics when reporting the results concerning the stimulus modality or pre-conditioning state of the subjects.
(2) The used pre-processing method to smooth out and clean the measured pupil raw data.
(3) The used measurement hardware and software framework in collecting pupil data.

In order to minimize the influencing factors, there are actions in the research community to provide the essential tools for pupil research to lower the barrier of entering the topic and ensuring the comparability of future research. A major step in this direction was the work “Standards in Pupillography” by Kelbsch et al., which summarized the current knowledge on pupil behavior and defined recommendations to be considered by author groups when reporting pupil study results (Kelbsch et al., 2019). The standardization approach mainly dealt with the minimal set of metrics that authors need to specify in published research, allowing third parties to reproduce experiments when necessary. Regarding the topic of data pre-processing, the focus is on which methods should be used to detect and remove artificially induced pupil changes, caused by eye blinks and fast gaze jumps during pupil recording sessions. Ranging from catching artifacts to smoothing out the measured raw data, a large number of software libraries and guidelines exist that can assist researchers in carrying out such tasks (Pedrotti et al., 2011; Canver et al., 2014; Lemercier et al., 2014; Attard-Johnson et al., 2019; Kret and Sjak-Shie, 2019; van Rij et al., 2019).

The research area of pupil behavior benefits from the interdisciplinarity of the research groups, which is promoted by the provision of tools and predefined standardized methodologies. However, the pupillometry technique itself is a significant hurdle, since there are no standardized requirements or reliable end-to-end open-source systems for recording pupil data in high-precision experiments under laboratory conditions.

## The Issue of Pupillometry

Typically, a pupil measurement can be performed manually by using a double-pinhole pupillometer (Holladay, 1926) or photographs with a reference object (Crawford, 1936) or through an integrated eye-tracking system. A higher proportion of pupil behavior studies is conducted by using an eye-tracking system, as identifying the pupil region is often a necessary step before estimating the gaze position (Lee et al., 2012). Commercial eye trackers from Tobii Pro, Smart Eye Pro or Eyelink are common solutions, which are easy to set up and usable without a technical background but cost approximately between 5,000 and 40,000 euros (Hosp et al., 2020; Manuri et al., 2020). Purchasing a set of high-resolution professional industrial cameras costs about 200 to 600 euros, with which an optical accuracy of 0.01 mm/px or more could be achieved. Thus, the price gap from commercial products results from the integrated software and license fees.

Commercial systems rely on closed software, restricting thereby conclusions about the used pupil-tracking algorithms, which is essential for the reproducibility. Additionally, based on the authors’ best knowledge, there is no commercial eye-tracking system that states the accuracy of their measured pupil diameter in the datasheet nor is a manual validation possible, as their solutions’ primarily focus is on gaze tracking. Especially in studies where pupil diameter effects are in a range of 10^–2^ mm, a validation of the system’s pupil measurement accuracy through a reference object is desirable.

The open-source head-mounted eye tracker project by *Pupil Labs* (Kassner et al., 2014) is an alternative to fully commercialized solutions, allowing free head movements and experiments in natural environments where a classic remote eye-tracking set-up is not possible. However, we do not recommend this system for precise pupil measurement applications, due to the cameras’ positions which are highly off-axis, causing pupil foreshortening errors (Hayes and Petrov, 2016). Additionally, the absolute pupil diameter is calculated indirectly by a method from which conversion accuracy is not yet fully validated for pupil measurements. Therefore, the solution provided by *Pupil Labs* is more suitable for experiments in which only the relative pupil diameter is of interest.

Remote tracking systems, positioned on the optical axis of the eye, are better suited for reliable pupil measurements. Various published approaches provide isolated components to build a custom remote stereo camera system (Hiley et al., 2006; Long et al., 2007; Kumar et al., 2009; San Agustin et al., 2010), which is not always feasible for interdisciplinary research groups, leading to a preference for commercial solutions. However, a groundbreaking project called *EyeRecToo* by Santini et al. (2017) has taken the first steps in establishing the idea of a competitive open eye-tracking software suite, which even has the option of choosing between different state-of-the-art pupil detection algorithms. Unfortunately, the software is mainly designed for head-mounted eye trackers or webcams and the use-cases are not targeted for the experimental pipeline of pupil research under laboratory conditions. For instance, a stereo camera arrangement with extrinsic calibration and the subsequent validation of a camera’s accuracy is not possible, to our best knowledge. Additionally, the software does not offer the option for evaluating external captured images from a stereo or mono camera system.

The success of the *Pupil Labs* project shows that end-users wish to have a fully integrated system consisting of hardware and software, packed with the functionalities of a professional commercial solution. Thus, in developing our proposed platform, we have focused not only on the functionalities and requirements of pupil researchers but also on the end-user’s experience, which should provide an easy way to build and run a pupil measurement system.

## Choosing Pupil Detection Algorithms for PupilEXT

The main application for an eye-tracking system is the estimation of a subject’s gaze location, which usually needs to recognize the pupil contour and its center position. Due to the high contrast between the sclera and the pupil region in a digital image, the recognition of the pupil is in principle possible through a combination of thresholding, edge detection and morphological operations (Goñi et al., 2004; Keil et al., 2010; Topal et al., 2017). State-of-the-art pupil detection approaches have additional steps in the image processing pipeline, ensuring a more robust contour fit while having a high and accurate detection rate. Under laboratory conditions, eye images are mainly captured using a NIR light source to avoid cornea reflections of the ambient environment, leading to optimized pupil detection. However, accurate pupil detection is an essential step in eye-tracking systems since a flawed edge detection could have an impact on the performance of an eye tracker (Santini et al., 2018a). Therefore, pupil detection methods intended for eye-tracking systems can also be used for pupil measurement, if an algorithm features the detection of aperture sizes.

There are three different illumination set-ups proposed for capturing a series of eye images that need to be in line with the used pupil detection algorithm (Li et al., 2005). In the bright-pupil method, a NIR-light source is placed close to the optical axis of a camera, resulting in a positive contrast between the iris and pupil region (Hutchinson et al., 1989). Due to the retinal reflection of the illumination back to the camera, the pupil region appears brighter than the iris and sclera itself (Li et al., 2005). In the dark-pupil method, the light source is placed off-axis to the camera. Thus, the pupil appears as a dark spot surrounded by the brighter iris (negative contrast). A third method called the image-difference technique leverages the image difference between dark- and bright-pupil to extract the pupil’s contour. For this, one NIR illumination should be positioned close to the camera’s optical axis (NIR 1) and a second one off-axis (NIR 2). By synchronizing the illuminations’ flashing interval with the sampling rate of a camera, one positive contrast image can be captured in a first frame (NIR 1, ON; NIR 2, OFF) and a second frame with negative contrast (NIR 1, OFF; NIR 2, ON). This approach can lead to a more robust pupil detection but has the drawback that more effort has to be invested in the illumination. Furthermore, two frames are needed for each captured pupil size value, reducing the overall sampling rate. The recent work of Ebisawa (1994, 2004), Morimoto et al. (2002), and Hiley et al. (2006) used this image-difference technique.

However, the core of a pupil measurement system is the algorithm that is used to determine the pupil diameter. Recently published works developed state-of-the-art approaches that can be applied in our proposed software *PupilEXT*. Similar to the work of Topal et al. (2017), we conducted a meta-analysis of 35 published pupil detection methods (Table 1) to evaluate and select suitable algorithms for our proposed measurement platform.

**TABLE 1:** Comparison of the pupil detection algorithms identified in the literature.

Algorithm	Approach basis	Downscaling	Bright/dark pupil	Thresholding	Ellipse fitting	Center of mass	Temporal information	Runtime in ms	Pupil size output	Blink detection	Confidence measure	Implementation available	Pupil size evaluation
Ebisawa, 1994	Image-diff.		⊙	🌑		🌑							
Zhu et al., 1999	Curvature		■	🌑	LSM	🌑			🌑				
Morimoto et al., 2000	Image-diff.		⊙	🌑		🌑	🌑	67					
Pérez et al., 2003	Threshold		■	🌑		🌑		40	🌑				
Lin et al., 2003	Edge		■		LSM	🌑		166	🌑				
Goñi et al., 2004	Threshold		□	🌑		🌑	🌑	33					
Ebisawa, 2004	Image-diff.		⊙	*		🌑	🌑	20		🌑			
*Starburst* Li et al., 2005	Rays		■	*	RANSAC		🌑	100^(3)^	🌑	🌑	○	🌑	
Hiley et al., 2006	Image-diff.		■	🌑		🌑		12^(2)^					
Long et al., 2007	Threshold	○	■	🌑		🌑^(1)^		6.67					
Dey and Samanta, 2007	Threshold	🌑	■	*	Circle			127	🌑				🌑
San Agustin et al., 2010	Threshold		■	🌑	RANSAC	🌑			🌑			🌑	
Kumar et al., 2009	Edge		■	*	LSM	🌑			🌑	🌑			
Keil et al., 2010	Threshold		■	🌑		🌑		60					
Lin et al., 2010	Threshold	○	■	🌑	🌑	🌑^(1)^			🌑	○			
Lanatà et al., 2011	Threshold		■	🌑	LSM				🌑				
Świrski et al., 2012	Threshold		■	*	RANSAC	🌑		3.77	🌑			🌑	🌑
Schwarz et al., 2012	Threshold		■	🌑					🌑			🌑	
Świrski and Dodgson, 2013	3D model		■	*	RANSAC	🌑			🌑			🌑	🌑
Kassner et al., 2014	Edge		■	🌑	LSM			45^(3)^	🌑	🌑	🌑	🌑	🌑
Chen and Epps, 2014	Threshold		■	*	LSM			60^(2)^	🌑	🌑	○		🌑
*ExCuSe* (Fuhl et al., 2015)	Edge	🌑	■	🌑	LSM			7	🌑	🌑		🌑	
*SET* (Javadi et al., 2015)	Threshold		■	🌑	🌑	🌑		100	🌑			🌑	
*ElSe* (Fuhl et al., 2016a)	Edge	🌑	■	*	LSM	🌑		7	🌑	🌑	○	🌑	
*PupilNet* (Fuhl et al., 2016b)	CNN	○	■										
*APPD* (Topal et al., 2017)	Curvature		■		MSM			5.37	🌑	🌑		○	🌑
*PuRe* (Santini et al., 2018a)	Edge	🌑	■		LSM			5.17	🌑	🌑	🌑	🌑	
*PuReST* (Santini et al., 2018b)	Edge	🌑	■	*	LSM		🌑	1.88	🌑	🌑	🌑	🌑	
Li et al., 2018	Edge		■		LSM				🌑				
*DeepEye* (Vera-Olmos et al., 2018)	CNN		■	*		🌑		33^(4)^				🌑	
*FREDA* (Martinikorena et al., 2018)	Image-diff.		■		🌑			63^(2)^	○			🌑	
*CBF* (Fuhl et al., 2018b)	Feature-class.	🌑	■					6.8				🌑	
*BORE* (Fuhl et al., 2018a)	Edge		■		🌑			15	🌑			🌑	
*DeepVOG* (Yiu et al., 2019)	CNN	🌑	■	🌑	🌑			17^(4)^	🌑	🌑	🌑	🌑	🌑
Eivazi et al., 2019	CNN	🌑	■					8^(4)^	🌑				

*We classified the algorithms by their applied technique and properties. This meta-analysis is inspired by the approach of Topal et al. (2017). Only a small portion of the proposed algorithms was evaluated through the detected pupil size, as stated in the last column. Typically, the authors assessed the pupil center’s accuracy, as eye tracking was the main application of most algorithms. The algorithms in the gray marked rows are available in our proposed pupil measurements software PupilExt. ○ only partial, ■ dark pupil, □ bright pupil, ⊙ bright and dark pupil, ^∗^ adaptive thresholding, 🌑 yes. (1) Symmetric center of mass, (2) MATLAB implementation, (3) Python implementation, and (4) graphics processing unit (GPU) aided.*

The potential algorithms need to estimate the pupil size, as this is the main focus of this work. From the 35 evaluated algorithms, we can rule out 11 approaches since they are not able to output the pupil size (Table 1). We decided to consider only algorithms designed for dark-pupil detection, serving to more freedom in setting up the position of the NIR light source. Another criterion for the selection was the availability of the implementation since we started from the working hypothesis that published procedures with existing programming code are ready for practical applications. Since our graphical user interface (GUI) should offer real-time pupil detection, only C++-implemented approaches were of interest.

Based on these criteria and taking the algorithms’ recency into account, we selected a total of six pupil detection approaches for *PupilEXT*. First, we decided to use the robust *Starburst* algorithm by Li et al. (2005), which was considered as a standard approach in pupil detection for a long time, implemented in several works throughout the years. Furthermore, we added the algorithm by Świrski et al. (2012), *ExCuSe* by Fuhl et al. (2015), *ElSe* by Fuhl et al. (2016a), *PuReST* by Santini et al. (2018b) and *PuRe* by Santini et al. (2018a). The algorithms *ElSe*, *ExCuSe*, *PuRe* and *PuReST* are licensed for non-commercial use only. The pupil detection algorithm from Swirski et al. is licensed under MIT, and the *Starburst* algorithm under GNU GPL. More details about the licensing terms of the detection algorithms can be found on the project page of *PupilEXT*^1^.

We did not select pupil detection approaches based on neural networks (Mazziotti et al., 2021). Models such as *DeepEye* (Vera-Olmos et al., 2018) and *PupilNet* (Fuhl et al., 2016b, 2017) reveal promising results, but their computational complexity is still too high for real-time pupil measurement applications without special hardware.

The user has the option to choose between these state-of-the-art algorithms for pupil measurement in the proposed *PupilEXT* platform. Additionally, the algorithms’ parameter can be checked and adjusted in the user interface to increase the software-based measurement accuracy, if necessary. By default, the *PuRe* algorithm is selected because it is considered as a top performer and the number of parameters are relatively user-friendly, making it to a generalized procedure for different measurement settings (Santini et al., 2018a, b). While the algorithms are solely based on recent publications from various author groups, the interested readership is referred to the original works of the respective pupil detection methods or works that already reviewed the algorithms (Topal et al., 2017; Manuri et al., 2020).

## Hardware Set-Up of the Camera System

**FIGURE 3:**
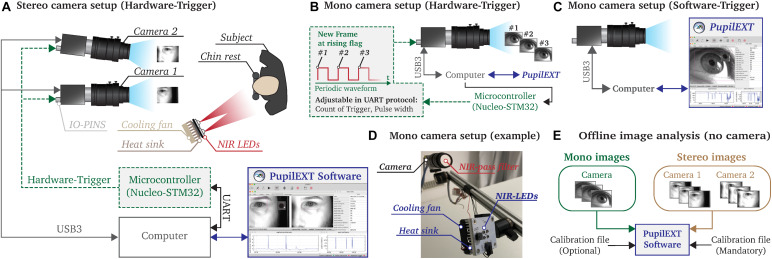
Illustration of the possible measurement configurations that can be realized with the *PupilExt* software. **(A)** In stereo vision mode, two cameras are connected to the computer via USB3.0. A microcontroller is connected to the IO-pins of the camera, which triggers a synchronized image acquisition on both cameras. We recommend using a Nucleo-STM32 microcontroller since the provided source code can be used to flash the electronic. We implemented a UART communication protocol in the microcontroller so that *PupilExt* can automatically control and synchronize the stereo camera system via the connected hardware, via the electronics. **(B)** In mono camera vision mode, it is possible to control the image acquisition by an external hardware trigger, which has the advantage that the recording time can be set accurately. When capturing multi-image sequences, the hardware trigger consists of a square wave signal in which each rising edge triggers an image acquisition. **(C)** The use of a microcontroller is optional when connecting a single camera. Without the use of a microcontroller, a software trigger is used for image acquisition. **(D)** Prototype of a pupillometry set-up with a single camera and respective near-infrared (NIR) illumination unit. **(E)** The software *PupilExt* can be used without connected cameras in an offline mode to detect the pupil diameter from externally captured images.

We linked the *PupilEXT* software with a specific camera brand (Basler) to provide a comprehensive platform for pupillometry. In this way, we allow a plug-and-play usage of the proposed system since the software is adapted to the hardware. The Pylon SDK is used to interface the cameras with the measurement software *PupilEXT*. Thus, any Basler branded industrial camera is integrable into the pupillometry platform. We explicitly do not support consumer webcams since *PupilEXT* is intended for reliable and accurate research applications. Generally, live or post-acquisition pupil measurements are supported through different measurement configurations (Figure 3).

Two cameras are needed for the stereo camera arrangement to detect the absolute pupil diameter directly (Figure 3A). One essential factor in the processing accuracy of such a configuration is the synchronization level between the cameras. Therefore, we synchronized the cameras through an external hardware trigger, leading to a stable system comparable with a professional manufactured commercial solution. Such a hardware trigger is needed to acquire images from both cameras simultaneously. In low-budget systems, the image acquisition is usually made by a software trigger that cannot guarantee synchronized image acquisitions, leading to reduced measurement accuracy. In our proposed system, the trigger signal is generated through a microcontroller, which is automatically controlled by *PupilEXT*. Additionally, we support pupil measurements with a single camera (Figures 3B–D). Here, the integration of a microcontroller for triggering an image acquisition is optional (Figure 3B). However, by including a microcontroller in the one-camera set-up, the duration of a recording session can be set. Note that when using a single camera, the pupil diameter is measured in pixels. Through an extra recording trial with a reference object, the pixel values can be manually converted to millimeters. If cameras are connected to *PupilEXT*, a real-time pupil measurement with one of the six pupil detection algorithms can be carried out. Furthermore, we support the option of recording images without pupil detection. In this way, it is possible to analyze the images in a post-acquisition mode without connected cameras (Figure 3E). In such an offline mode, image sequences from externally recorded cameras can also be loaded, making it possible to leverage the software on already existing pupil image datasets.

We recommend a NIR illumination unit to avoid corneal light reflections in the eye from the visible spectrum, which could impact the accuracy of pupil detection. For this, a NIR bandpass filter should be mounted in front of the camera’s lens. The advantage of a NIR-based measurement is that the image quality does not suffer in pupil light response experiments. Both the source code of the microcontroller for generating the hardware trigger and the respective NIR circuit board design (Figure 3D) are provided together with the *PupilEXT* software, allowing to set up the system effortlessly. The following subsections deal with the different operational configurations of the platform (Figure 3) and the needed hardware elements in more detail, ensuring the reproducibility of the measurement platform.

### Camera Set-Up

We built a prototype consisting of two Basler acA2040-120um cameras with 50-mm lenses to validate the pupillometry platform in a sample study. The cameras operated in stereo vision mode to measure the absolute pupil diameter. The cameras support a resolution of 2,048 px × 1,536 px with a maximal frame rate of 120 fps. We positioned the system in front of an observer at a working distance of 700 mm, with a baseline distance between the cameras of 75 mm in which the secondary camera has an angle of 8° to the main camera (Figure 3A). A NIR illumination unit, consisting of four LEDs with a peak wavelength of 850 nm (SFH-4715AS), is placed near the subject’s head without obstructing the view of the cameras. Furthermore, the camera lenses are equipped with a high-pass infrared filter (Schneider IF 092 SH) with a transmission range of 747 to 2,000 nm, which should reduce artifacts from the ambient illumination.

The cameras are connected through their USB 3.0 interface with the computer for data transmission. Additionally, the IO-Pin connector of the cameras is used to adjust the timing, execution and synchronization of the image capturing. A microcontroller (Nucleo STM32F767ZI) is integrated into the pupillometry platform, controlling the cameras’ capturing interval through a shared digital signal.

For this, the microcontroller transmits a periodic square waveform modulated signal with a voltage amplitude of 3.3 V. Each rising edge of the signal triggers an image (Figure 3B). The frequency and duration of the square wave signal are adjustable through *PupilEXT*, affecting the frame rate and recording time of the camera. While the use of a microcontroller is obligatory when shooting stereo vision, it can be used optionally in the single-camera set-up (Figures 3B,C). Before an absolute pupil measurement can be carried out in stereo vision mode, extrinsic and intrinsic calibrations of the cameras need to be performed in *PupilEXT*.

### Embedded Hardware Trigger

In stereo vision mode, the microcontroller must be connected to the computer so that *PupilEXT* can communicate with the embedded electronic via UART. We have implemented a simple text-based protocol in the microcontroller, for starting and stopping the trigger signal. Control commands can be dispatched via the graphical interface in *PupilEXT* or manually through a serial port terminal application like *CoolTerm* or *HTerm*. If the provided embedded microcontroller source code is not used, users can easily implement the protocol themselves in their preferred microcontroller brand.

To start a trigger signal, the parameters *COUNT_OF_TRIGGER* and *TIME_TRIGGER_ON* must be set in the protocol. The parameter *COUNT_OF_TRIGGER* indicates how many rising flags should be transmitted. The parameter *TIME_TRIGGER_ON* sets the pulse width in microseconds, which is used to set the sampling rate of the camera. Both parameters are set with the string command *< TxCOUNT_OF_TRIGGERxTIME_TRIGGER_ ON >* via the UART interface of the microcontroller. The “x” term is used as a separator between the parameters. For instance, if a trigger signal should be used for capturing a total of 100 images with a rate of 10 ms, the protocol would correspond to *< Tx100x5000 >*. A detailed introduction of how to flash and install the embedded electronic is provided on the project’s webpage.

## The Cross-Platform Software Suite

The core of the pupillometry platform consists of the software *PupilEXT*, structured and implemented based on the requirements of scientifically oriented pupil behavior research. Although pupil measurements can be performed with commercial eye-tracking solutions, the closed system design blocks the transparency of used pupil detection algorithm and the determination of its pupil measurement accuracy. Moreover, such commercial systems are not fully intended for absolute pupil measurements. With *PupilEXT*, we offer not only a free alternative to commercial solutions but also extended features in the topics of pupil detection, measurement resolution, data acquisition, image acquisition, offline measurement, camera calibration, stereo vision, data visualization and system independence, all combined in a single open-source interface.

**FIGURE 4:**
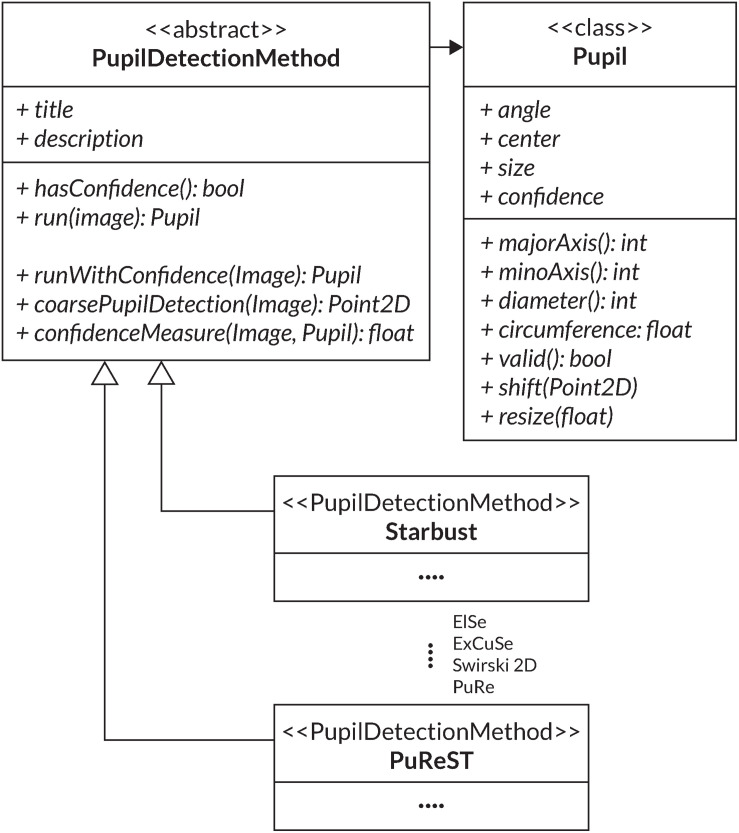
UML diagram of the *PupilDetectionMethod* interface used to implement the various pupil detection algorithms. Additionally, the Pupil class is used for collecting a pupil detection result.

It is possible to choose between the six discussed pupil algorithms (*Starburst*, *Swirski*, *ExCuSe*, *ElSe*, *PuRe* and *PuReST*) and to freely adjust their processing parameters and to optimize the pupil contour’s detection accuracy. Additionally, the parameters of a pupil detection method can be reported, leading to an increase in the reproducibility of pupil examinations. We have integrated the pupil detection methods into one unified framework by using a standard pupil detection interface (Figure 4).

For this, the *PupilDetectionMethod* interface is adapted from the *EyeRecToo* eye-tracking software (Santini et al., 2017), which employs an interface to integrate multiple pupil detection algorithms. It defines a set of abstract methods like *run* and *hasConfidence*, which are concretized through the specific algorithm implementation (Santini et al., 2017). The *run* method defines the respective pupil detection algorithm that returns a detected pupil from an image. Through *hasConfidence*, we verify the availability of a confidence measure from a respective algorithm. The interface provides a general confidence measure that can be used if an algorithm does not provide its confidence measure (Santini et al., 2018a). An additional component that is adapted from *EyeRecToo* (Santini et al., 2017) is the *Pupil* class, which aggregates all data of a detected pupil and its fitted ellipse into one class. A simplified UML diagram of the adapted structure is illustrated in Figure 4.

In *PupilEXT*, the camera frame rate is adjustable up to 120 Hz. Pupil measurement data are stored in a comma-separated value (CSV) file containing the pupil diameter, confidence measure and ellipse parameters. Besides recording real-time pupil data, the software features storage of raw images for later pupil evaluation. A comprehensive stereo and mono calibration procedure within the software guarantees an accurate and validatable measurement pipeline. The unique feature is the integration of professional industrial cameras with stereo vision capabilities, dedicated to absolute pupil diameter measurements. Metrics are visualized in real-time during pupil measurements, providing an *ad-hoc* evaluation of metrics.

### Camera Interface

Before *PupilEXT* can perform a remote pupil detection, images must be grabbed from the camera(s). We access the Basler cameras with their USB 3.0 interface using a manufacturer-provided programming library called *Pylon*. Through the library, we configure both the camera preferences and activate an image capturing trigger for passing to the image processing pipeline. We distinguish between two image acquisition modes of a camera. With a software trigger, the camera acquisition is controlled over the *Pylon* library interface to record images at a specified frame rate continuously. In the single-camera mode, commonly, the software trigger is used, and the hardware trigger is optional. The hardware trigger is mainly implemented for the stereo vision mode, in which two cameras synchronously capture images upon a receiving a signal flag on an IO-pin. In stereo camera set-ups, the integration of the hardware trigger is obligatory. In such set-ups, a software trigger cannot guarantee that both cameras capture an image at the same time, affecting the performance of a stereo system. Connection establishment and message transmission to the microcontroller is accomplished via a serial port. The microcontroller configuration includes the settings for a camera frame rate as well as the duration of the recording.

**FIGURE 5:**
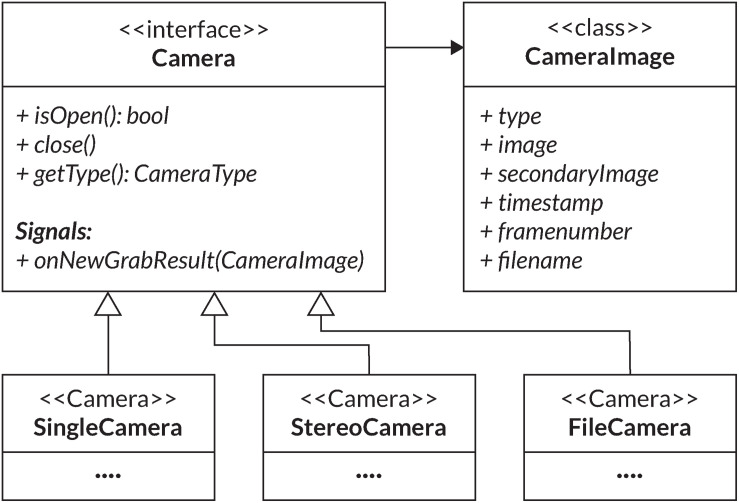
UML diagram of the Camera interface and its implementations modeling for different types of cameras in the *PupilEXT* software. The *CameraImage* class is used to represent resulting images and their corresponding metadata.

To integrate the camera(s) in *PupilEXT*, a *Camera* interface was created, defining a set of functions for all camera types (Figure 5). Three types of cameras are differentiated: a single camera, a file camera and a stereo camera consisting of the main and secondary cameras (Figure 5). The file camera can be viewed as a camera emulation used in offline pupil detection sessions from previously recorded images retrieved from disk storage. However, by emulating the playback of images as a camera object, it can be integrated seamlessly into existing functions of *PupilEXT*. For the representation of the camera image, the *CameraImage* class is defined (Figure 4). The image distribution in the *PupilEXT* software from the camera(s) is organized with an internal event handler function. For this, the *Pylon* camera library provides an interface that is called every time a corresponding camera acquires a new image. However, for a stereo camera set-up, an image recording consists of two corresponding images that will be delivered by two separate function calls.

The initial approach was to leverage a camera internal timestamp to associate the two corresponding images. However, matching the two cameras, internal timestamps of corresponding images led to a buggy image rectification. Therefore, it was necessary to find a more reliable approach. Besides the camera(s) internal timestamp, additional metadata such internal frame count is provided by the *Pylon* API. As long as both cameras start the acquisition simultaneously, the frame counts match. This approach ensures a fixed and reliable order of stereo image acquisitions processed by *PupilEXT*.

### Image Recording and Reading for Offline Analysis

For retrospective detection of the pupil diameter, raw image sequences from the camera can be stored directly on the hard disk. Here, a decision about the format of the images needs to be made. Users can choose between Windows Bitmap (BMP), Tagged Image File Format (TIFF) and JPEG in the preferences of *PupilEXT*. The BMP format represents an uncompressed image format, resulting in large file size. In contrast, JPEG is a lossy compressed format commonly used in consumer photography due to its small size. The TIFF cannot be directly categorized into either of these classes, as it represents an adaptable container that can hold both compressed and uncompressed image formats. A clear-cut decision on which format to use cannot be made easily. While uncompressed formats such as BMP would result in the highest quality of images, the size of data that needs to be handled cannot be underestimated. For the use case of recording images on a disk, one needs to be able to write image data with a rate up to the camera’s maximal frame rate, i.e., 120 fps.

Given the camera(s) of the reference system with a resolution of 2,048 px × 1,536 px and assuming a bit depth of 8 bits for greyscale images, the resulting image size is ≈3.15 MB. However, with 120 images per seconds, this results in a required writing speed of ≈377.49 MB/s for a single camera and ≈755 MB/s for the stereo set-up. Image size for compressed formats such as JPEG cannot be estimated this easily. Thus, an average image size observed from sample recordings of the reference system is taken. Results are greyscale images with an average size of around 840 kB. Consequently, JPEG requires a writing speed of up to ≈100 MB/s for a single camera and around 200 MB/s in a stereo camera setting. Solely based on the required writing speed without incorporating delays from, i.e., the computational overhead of compression, the speed of traditional hard disk drives (HDDs) is only sufficient for writing JPEG images in a single-camera set-up. More modern hardware in form of SATA 3, solid-state drives (SSDs) can further handle single and stereo camera set-ups for JPEG images, or just a single camera using BMP images. For recent NVMe-based SSDs, the writing speed is theoretically sufficient for writing BMP images in a stereo camera set-up. Note that the discussed rates all referred to the maximal frame rate of 120 fps. Saving images for later analysis is generally recommended for short recordings where the accuracy of the various pupil detection algorithms is of interest.

### Pupil Diameter Recording

Pupil data are recorded in CSV files that store all acquired values of a pupil measurement. Pupil values can be recorded in an online measurement with connected cameras or in an offline measurement in which images are loaded in *PupilEXT* for post-acquisition evaluation. For online measurements, each pupil measurement is associated with a timestamp provided by the system time in milliseconds since Unix epoch, which is synchronized with the camera’s internal hardware clock. In offline measurements, where images are read from files, no timestamp is available. Thus, the corresponding filename is used to associate each measurement. The fitted ellipse can be reconstructed from the stored ellipse parameters: width, height, center position and angle. Further recorded data for analysis are the pupil diameter, circumference and confidence measure. The pupil diameter is stated in pixel by default, and when in stereo mode, it is additionally stated in absolute units.

Regarding the pupil detection confidence, a value is only available when the applied pupil detection algorithm provides such a measure. However, a second confidence value called outline confidence is provided independently of the used algorithm. This confidence measure is based on the outline contrast of the inner and outer regions of the fitted ellipse (Santini et al., 2018a). The goal of such value is to describe the reliability of the detected pupil diameter. These measures are useful to directly filter pupil detections that may constitute a false detection or include high uncertainty. Filtering out such detections is a common practice in the pre-processing of pupil detection results (Kret and Sjak-Shie, 2019). Santini et al. (2018a, b) apply a combination of different metrics for their confidence measure. Besides the outline confidence, the ellipse axis ratio and an angular edge spread metric are used. The ellipse axis ratio describes the ratio between major and minor axes of the ellipse, aiming to state the degree of distortion of pupil fit. The angular edge spread measures the spread of the found points on the fitted ellipse. If the points are evenly distributed, it is more likely that they originate from an exact pupil contour. We simplified the accessibility of the data by using a tabular text-based format, i.e., in the form of a CSV file. This format is independent on the used system and is commonly used for measurement recordings.

### Camera Calibration

The goal of the camera calibration is to remove distortions caused by the camera lens and to estimate a projective transformation for mapping world coordinates to image coordinates. A camera projection matrix in the form of *M* = *K*[*R*⋅*T*] is used for mapping. *K* denotes the intrinsic parameter and *R*⋅*T* the extrinsic parameter matrices. The intrinsic matrix *K* projects points in the camera coordinate system to the image coordinate system with the values of the focal lengths (*f*_*x*_, *f*_*y*_) and the optical center (*c*_*x*_, *c*_*y*_) of a camera. These parameters are independent on the viewed scene and are reusable. The extrinsic matrix [*R*⋅*T*] represents the projection of world coordinates to camera coordinates, consisting of a 3 × 3 rotation matrix *R* and the 3 × 1 translation vector *T* (OpenCV, 2020). By using the camera projection matrix M, an image coordinate *P_c_* can be projected into the associated world coordinates *P_W_*. Such projection is typically applied in stereo vision, where the camera matrices of two or more cameras are used to estimate the depth and position of a point in world coordinates captured by these cameras. A further application of camera calibration is the correction of lens-induced distortion. Here, two types of distortion exist, radial and tangential distortions. For correcting distortions in a pinhole camera model, the calibration process estimates coefficients representing the distortions in the image, resulting in the five distortion coefficients *C* = (*k*_1_, *k*_2_, *p*_1_, *p*_2_, *k*_3_).

#### Implementing Single-Camera Calibration

In *PupilEXT*, we perform the single-camera calibration, e.g., the estimation of the camera parameters *K* with the computer vision library *OpenCV* library and its calibration routines. For this, a total of 30 images are collected with a rate of 0.5 fps, independently from the adjusted camera frame rate. After one image is collected, the depicted calibration pattern is detected, and feature points of the pattern were extracted. Successfully detected feature points and their positions are then stored and visualized in the calibration interface of *PupilEXT*. If the detection was not successful, the image is discarded, and the process will be applied again to the next camera image. This procedure is repeated until the specified number of images is collected. The camera calibration process is performed when enough feature points are collected. This function optimizes the camera parameters by minimizing the reprojection error according to the algorithm of Zhan (Zhang, 2000). The reprojection error describes the root mean square error (RMSE) distance between the reprojection of the observed feature points using the current camera parameters and their known position in the calibration pattern.

After successful camera calibration, the quality of the resulting calibration is an essential metric. Its quality is primarily dependent on the accuracy of the detected feature points, which is an edge detection task similar to pupil detection. We report in the *PupilEXT* interface the final reprojection error in the form of the RMSE. However, as this error constitutes a mean squared distance, it may be less intuitive for the user. Therefore, we compute an additional error using the mean absolute error (MAE), measuring the arithmetic mean of the absolute distances between the observed feature points and their projected estimation. The reprojection procedure of the MAE distance is identical to the reprojection error returned by the calibration routine. A set of ideal feature points of the calibration pattern in world coordinates are projected into the image plane using the estimated intrinsic and extrinsic parameters *K*, *R* and *T*. After the projection of the ideal feature point positions into their estimated image coordinates, they can be compared with the actual detected feature points in the captured image. The deviation is stated in the form of the Euclidian distance between the detected and idealized point positions, describing how well the camera parameter approximates the actual camera projection.

#### Validate Single-Camera Calibration

The reported reprojection error is based on the camera’s projection matrix, optimized for the collected set of images during calibration. Therefore, the reprojection error may contain a bias due to overfitting. For quantifying potential overfitting, an additional verification feature is implemented in *PupilEXT*, performing the same procedure as in the calibration step but using fixed camera parameters. For this, we capture new calibration pattern images during the verification and calculate the reprojection error again, representing an unbiased approximation of the calibration quality. For instance, our prototyped single-camera system (Figure 3D) achieved an RMSE reprojection error of 0.341 px, where values under one pixel are commonly referred to as good calibration quality. For the MAE reprojection error, we achieved a value of 0.041 px, meaning that the average feature point coordinate was projected into the image plane with such a distance error. The verification with a new set of images showed a MAE reprojection error of 0.040 px.

In *PupilEXT*, the calibration parameters are stored to support the reuse at a later point. For this, a configuration file is saved after a successful calibration is completed. The file contains all essential information to reproduce and assess the state of the camera calibration, such as the attributes of the calibration pattern, the estimated camera parameter matrices and all projection error measures. The functionality of saving and restoring the calibration configuration enables an additional use case, the correction of image distortions in offline pupil measurements.

### Stereo Camera Calibration

**FIGURE 6:**
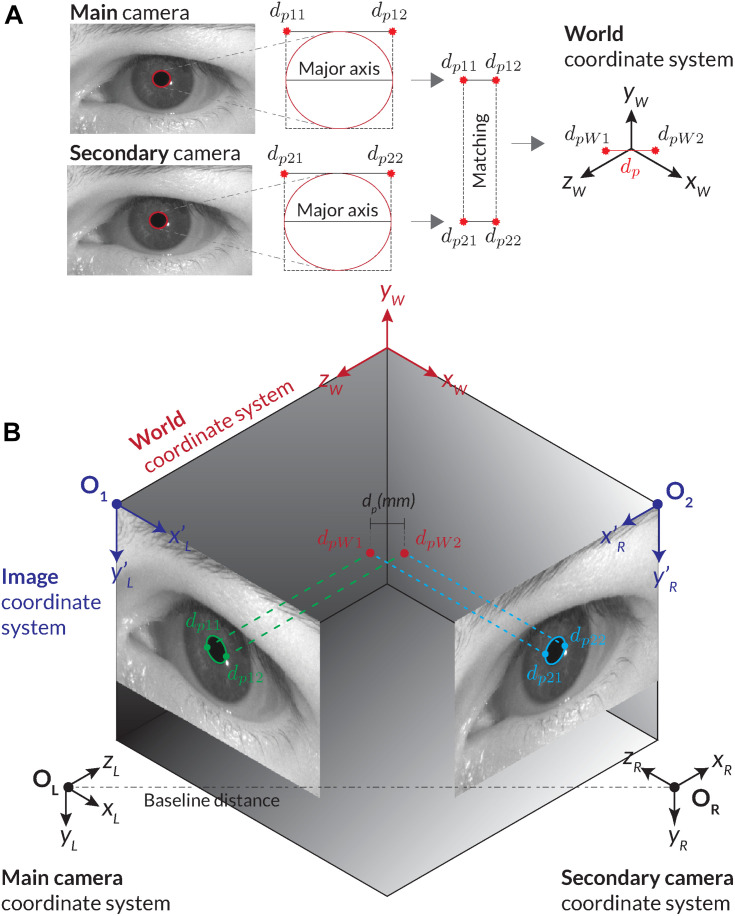
Illustration of calculating the absolute pupil diameter with the stereo vision set-up. **(A)** For the corresponding stereo images, two pupil detections are carried out. We use the ellipses of the pupil detections and their minimal encompassing rectangle as feature points for matching. Through triangulation, the corresponding stereo images are transformed into world coordinates. The absolute pupil diameter is calculated with the Euclidian distance between the two world coordinates. **(B)** Procedure of the stereo transformation with the main and second cameras’ images.

Stereo vision offers the possibility of tracking the depth information and absolute pupil size from two or more images captured by cameras of known position. By using the calibration matrices *M_i_* of two cameras, it is possible to triangulate image coordinates in both images to their corresponding world coordinates *P_W_*. For this, matched points in both images must be found. Therefore, the pupil detection must be applied to images from both cameras (Figure 6A). For absolute pupil size calculation, the ends of the major axis of the ellipse are extracted and triangulated into world coordinates, and their distance was computed through the Euclidian distance (Figure 6A).

Triangulation determines the world position of an image point through its perspective projection in two or more images. Each projection point in an image corresponds to a projection line in world coordinates, representing all possible world coordinate positions that could have projected this point into the image. The projection lines of corresponding points can be used to determine their intersection in world coordinates. Figure 6B shows two corresponding image points of the main and secondary cameras (*d*_*p*11_, *d*_*p*21_) and their intersection point *d*_*pW1*_ in the world coordinate system. There are two challenges with this approach. First, the corresponding pupil detections in both images are required to retrieve matching points.

Second, extraction of feature points from a pupil contour may be ambiguous due to blurriness of the edge. If an identical pupil detection in both images cannot be guaranteed, potential deviations can be prevented by detecting and filtering those situations from the data stream. In *PupilEXT*, we use the corners of the minimal encompassing rectangle of the fitted ellipse (*d*_*p*11_, *d*_*p*21_) and (*d*_*p*21_, *d*_*p*22_) as feature points for triangulation (Figure 6A). The corner points correspond to the major axis of the ellipse for having a more robust feature selection in both images.

#### Implementation of Stereo Vision

Given the two recognized pupil ellipse results from the main and second cameras (Figure 6B), we check the success of pupil detection and confidence in both images. Naturally, if one of the detections failed, no matching points (Figure 6A) can be extracted or triangulated into the world coordinate system. In valid cases, the feature points (*d*_*p*11_, *d*_*p*12_) and (*d*_*p*21_, *d*_*p*22_) of both ellipse fits are extracted. Here, the bounding rectangle of the ellipse fit is leveraged, and the corner points from the major axis are extracted (Figure 6A).

Assuming the calibration parameters of both cameras are available, the paired ellipse image point coordinates (*d*_*p*11_, *d*_*p*12_) and (*d*_*p*21_, *d*_*p*22_) are corrected for potential distortions using the distortion coefficient matrices. Next, the corresponding image feature points (*d*_*p*11_, *d*_*p*21_) and (*d*_*p*12_, *d*_*p*22_) are triangulated using the OpenCV function *cv::triangulatePoints*. The triangulation results *P*_*H*1_ and *P*_*H*2_ are represented in homogeneous coordinates, which then are converted into Cartesian coordinates (Eqs. 1 and 2).

(1) $$P_{H}=\left[\begin{array}{c} X_{H}\\ Y_{H}\\ Z_{H}\\ W_{H} \end{array}\right],\mathrm{home2cart}\left(P_{H}\right)=\left[\begin{array}{c} X_{H}/\omega \\ Y_{H}/\omega \\ Z_{H}/\omega \end{array}\right]$$

(2) $$\omega =\left\{ \begin{array}{cc} W_{H}, & \mathrm{if}W_{H}\neq 0\\ 1, & \mathrm{otherwise} \end{array}\right.$$

With the transformed points in the world coordinate system (*d*_*pW*1_, *d*_*pW*2_), we determine the absolute pupil diameter through the Euclidian distance (Figure 6B). In the experiments, the computation time of this procedure (feature extraction, distortion correction and triangulation) was on average 0.03 ms, which should not significantly influence the maximum possible processing rate of pupil measurements.

However, no further criteria are applied for checking the reliability of the stereo vision result, as it is left open for the user applying the post-processing procedure. We did not consider a general threshold for pre-filtering to be necessary since the user should have full control over the evaluation of the data. For this, we provide all necessary raw data from both cameras in the recorded CSV file.

#### Calibration of Stereo Vision

A requirement for the stereo triangulation is the projection matrices *M_i_* of both cameras. As discussed in the *Camera Calibration* section, the parameters of a single camera are estimated in the calibration procedure, resulting in the intrinsic parameters of the cameras. As the projection matrix *M* consists of both the intrinsic and extrinsic parameters, the extrinsic parameters are estimated through a OpenCV stereo calibration procedure, which takes the intrinsic parameters of each camera, returning the extrinsic parameter in the form of the rotation matrix *R* and the translation matrix *T*. Thereby (*R*, *T*) describe the relative position and orientation of the main camera with respect to the secondary camera coordinate system (OpenCV, 2020). After the estimation of these extrinsic parameters, the projecting matrices *M*_1_, *M*_2_ can be calculated with the equation *M* = *K*[*R*⋅*T*]. Notably, in a stereo camera set-up, the main camera is typically selected as the origin of the stereo camera coordinate system. Thus, the projection matrix of the main camera does not apply rotation or translation and is therefore given by *M*_1_ = *K*⋅[*I*|0], where *T* is replaced with the identity matrix *I* and *R* is replaced with the zero vector.

#### Validate Quality of Calibration

Similar to the single-camera calibration, the reprojection error is returned as RMSE by the stereo calibration procedure. In stereo vision mode, the reprojection error states the distance between the observed and reprojected feature points combined for both cameras in image coordinates. However, for the user, it would be more useful to be able to assess the quality of the stereo calibration in terms of absolute units. Therefore, we leveraged the predefined size of the calibration pattern to calculate the measurement error of the calibration in absolute units. For this, we measure the absolute square size of, i.e., the chessboard pattern, using the detected feature points from both cameras in the calibration routine. The detected feature points of the calibration pattern are undistorted, stereo triangulated and converted into Cartesian world coordinates.

Next, the measured square size is compared with the known distance between two corner feature points of the calibration pattern. As a result, we report the calculated error of the stereo camera system in absolute units calculated by the distance deviation between the measured and idealized sizes of the pattern. However, the stated error again could be biased by the overfitting in the calibration routine. Therefore, we implemented a verification routine that checks the absolute measurement error using a new set of images with the calculated projection matrices. Similar to the single-camera mode, the stereo calibration matrix can be saved and loaded into the software for the next usage, reducing new calibration effort. Here, we recommend verifying the old calibration before a pupil measurement is conducted. If the lens settings or camera position are slightly changed, the transformation matrix needs to be re-created by a new calibration procedure. The necessity can be quickly checked using the verification function in *PupilEXT*.

### Performance of PupilEXT

The performance of *PupilEXT* in pupil measurements depends on various factors such as processing power of the system, frame rate of the camera and the applied pupil detection algorithm. As listed in Table 1, the runtimes of the pupil detection algorithms vary significantly. For the goal of conducting pupil measurements with a frame rate of 120 fps, a maximal runtime of around 8 ms or less is necessary. Additional computations such as correcting lens distortion can increase the needed computation time per image. We optimize the computational complexity in *PupilEXT* by using a region of interest (ROI), reducing the amount of pixel that needs to be processed. The ROI can be adjusted interactively by the user in the interface.

In combination with the *PuRe* pupil detection algorithm, we achieved a stable pupil measurement at 120 fps on full images. With manually specified ROI selection, the frame rate can be pushed further, as *PupilEXT* is completely implemented in C++, supported by parallel computation using CPU threads.

### The Graphical User Interface of PupilEXT

**FIGURE 7:**
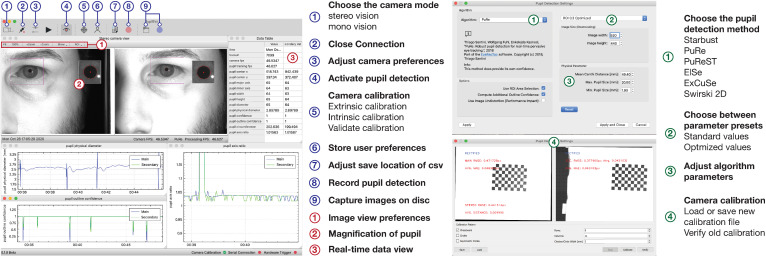
The graphical user interface of the programmed software *PupilEXT* during a pupil recording in stereo camera mode. In the main window’s taskbar, various functions can be accessed for quick actions. In the pupil detection sub-window, a pupil algorithm with respective parameters can be adjusted. The camera calibration can be done directly in *PupilEXT*. Calibration files can be saved and validated to give an outline of the camera system’s edge detection accuracy, which is essential for a pupil measurement pipeline.

Figure 7 illustrates the GUI of *PupilEXT* during a pupil measurement in the stereo camera mode. Via the taskbar of the GUI (Figure 7, points 1 to 9, blue), the essential function of the software is linked. Before a pupil measurement, the camera mode and the respective cameras must be selected to establish a connection (Figure 7, point 1, blue). In the camera settings also a connection to the microcontroller can be established if a hardware trigger is required. After successful connection to the cameras, a window with a live image view of the cameras is opened. Camera parameters such as gain factor, exposure time or maximum frame rate can be changed at any time via a quick start button (Figure 7, point 3, blue). Next, one of the six pupil algorithms can be selected in the pupil detection preferences (Figure 7, point 1, green). In addition to the algorithms, the parameters of the method can be set to optimize the detection accuracy when necessary (Figure 7, point 3, green). We have provided a preset of parameters that can be selected (Figure 7, point 2, green). In addition to the standard parameters from the original papers, we have added optimized values that are adapted to different ROI sizes. We have set the *PuRe* method as a standard method in *PupilEXT*.

The pupil detection of the captured live images can be started with the eye symbol in the main window (Figure 7, point 4, blue). We provided in the live view window a quick action menu (Figure 7, point 1, red), which can be used to adjust the image size, setting the ROI or displaying magnification of the pupil. The ROI features allow placement of a rectangular area over the eye to improve performance further when recordings at a higher frame rate of 120 Hz are needed. Note that for the stereo camera mode, a calibration should be carried out; otherwise, the absolute pupil diameter will not be available. The calibration window can be reached through the taskbar in the main window (Figure 6, point 5, blue).

In the calibration window (Figure 7, point 4, green), one can select the type of calibration pattern. Next, the calibration can be started, resulting in the calibration file that is saved locally on the hard disk. If a calibration file already exists, it can be loaded via the calibration window (Figure 7, point 4, green), and its validity can be again verified. The stated calibration accuracy can be recorded in a CSV file during the validation procedure.

After the calibration is completed, the absolute pupil diameter is displayed in the data view, which also lists all tracked pupil values in real-time (Figure 7, point 3, red). Each of these values can be visualized in a real-time plot by selecting the specific value in the data view. For recording the pupil measurements, a disk location can be selected to save the pupil data in a CSV file (Figure 7, point 7, blue). The data can be saved continuously with the recording button (Figure 7, point 8, blue). The raw images can be saved with the blue recording button (Figure 7, item 9, blue) for later offline pupil detection in *PupilEXT*. In the Supplementary Materials, we have added hands-on video materials to illustrate the pipeline of usage and the features. Additionally, we offer the feature of creating and loading custom profiles (Figure 7, point 6, blue), which opens the software in a specified state to avoid the workload when *PupilEXT* is started next time.

## Demonstration of a Measurement Pipeline With PupilExt

To illustrate the measuring procedure with *PupilEXT*, we performed an exemplary experiment on the wavelength-dependent pupil light response. We recorded the pupil diameter of an observer with six repetitions (trials) using *PupilEXT*, while different light spectra were turned on at a steady luminance. For this, a subject looked into a 700 mm × 700 mm sized homogeneously illuminated observation chamber. The illumination was generated by a custom-made temperature-controlled (30°C ± 0.1°C) multi-channel LED luminaire, which was used to trigger the pupil diameter with chromatic stimulus spectra (Zandi et al., 2020). Pupil foreshortening error (Hayes and Petrov, 2016) was minimized by using a chin rest for positioning the subject’s head. Additionally, the gaze point was fixed with a 0.8° sized fixation target (Thaler et al., 2013) in the middle of the adaptation area. On the left eye’s optical axis, a stereo camera system consisting of two Basler acA2040-120um cameras with 50-mm lenses was set up (Figure 3A).

The pupil diameter was triggered using chromatic LED spectra with peak wavelengths **λ_**P***eak*_** of 450 nm [full width at half maximum (FWHM): 18 nm, **L** = 100.4 cd/m^2^ ± SD 0.23 cd/m^2^) and 630 nm (FWHM: 16 nm, **L** = 101 cd/m^2^ ± SD 0.31 cd/m^2^], which were switched on for 30 s. Before each stimulus spectrum, a phosphor-converted white-colored LED with a correlated color temperature of 5,500 K (**L** = 201 cd/m^2^ ± SD 0.48 cd/m^2^) was presented to adapt the pupil diameter to its baseline. The order of the chromatic stimulus spectra was randomized. One pupil measurement trial lasted 240 s, as the anchor spectrum (5,500 K) was switched on twice between each chromatic stimulus for 90 s, and the main stimuli (450 and 630 nm) were switched on 30 s. The spectra were measured 20 times before and after the experiment using a Konica Minolta CS2000 spectroradiometer. We controlled the luminaire with a custom-made MATLAB script, which stored the switch-on times of the spectra in a CSV-File. Possible switch-on latency times during the command transmission from MATLAB to the luminaire’s hardware were taken into account by tracking the processing time in the embedded software. We recorded stereo eye images with 30 fps (^∗^.bmp) during each pupil examination trial (240 s), making it possible to detect the pupil diameter from the images with different detection algorithms, later on using the offline pupil analysis mode of *PupilEXT*. The pupil data were synchronized with the luminaire’s switch-on times afterward using a MATLAB script.

### Pre-processing the Measured Raw Data

**FIGURE 8:**
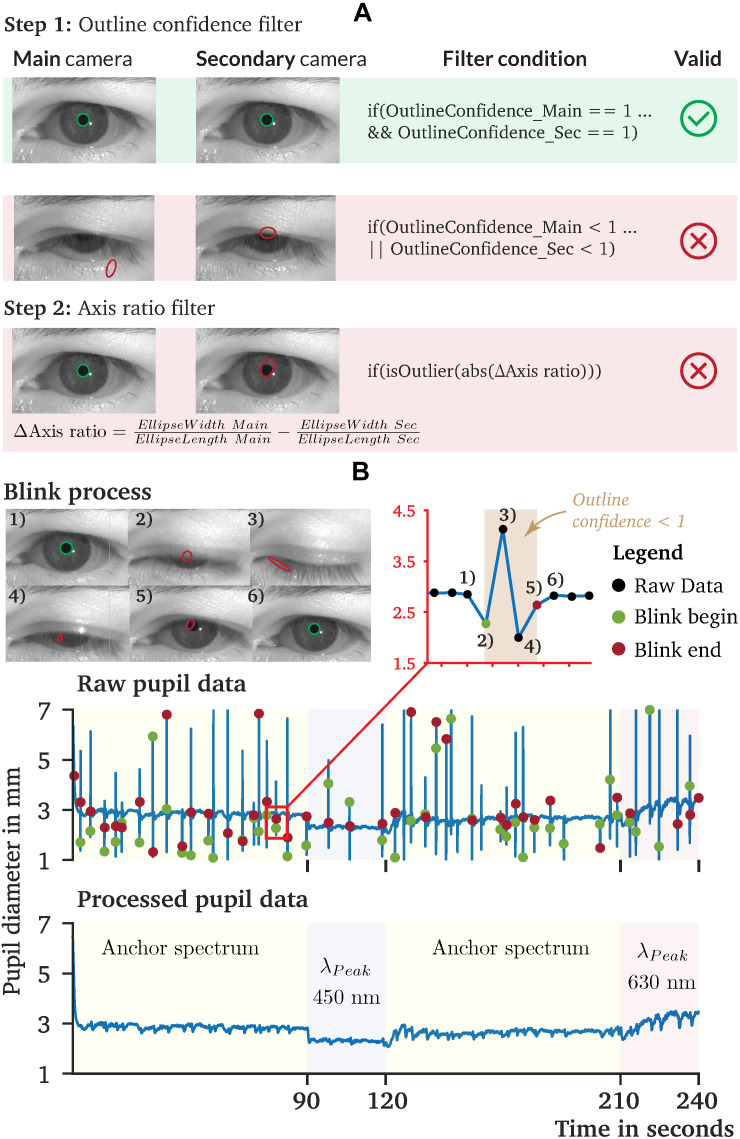
Recorded pupil data and proposed pre-processing procedure of pupil diameter data collected by *PupilEXT*. **(A)** A two-step pre-processing procedure is proposed, which uses the outline confidence and the axis ratio of the cameras’ tracked ellipses. **(B)** Recorded pupil data from our sample experiment to illustrate the performance of *PupilEXT*. The outline confidence can be used to identify eye occlusions in the data approximately. The two-step pre-processing can remove artifacts and other unnatural physiological pupil diameter changes.

Recorded raw pupil data are usually occupied by artifacts or other non-physiological pupil changes that need to be pre-processed (Figure 8A). For the pupil data recorded by *PupilEXT*, we recommend a two-step filtering procedure. First, every data point that has an outline confidence measure (Santini et al., 2018a) lower than 1 should be left. With this step, artifacts caused by eye blinks are detected robustly (Figure 8A). Other artifacts can occur if the matching points (Figure 6B) between the first and second cameras differ, resulting in a non-physiological shift of the pupil diameter, visible through slight peaks in the data. We identify matching point errors by comparing the stated axis ratio of the ellipses between the main and second cameras. The axis ratios differ because of the second camera’s positioning causing a perspective pupil area change. However, the ellipse axis ratio difference between the ellipses of cameras 1 and 2 should remain constant within a certain range. Thus, the reliability of the matching points (Figure 6B) can be detected by calculating the difference of the axis ratio across the data points and removing all strong outliers from the sample dataset (Figure 8A). We have pre-processed the recorded pupil data according to this two-step procedure. The results of one raw pupil measurement trial (240 s) using the *PuRe* algorithm and respective pre-processed pupil data are shown in Figure 8B. Eye blinks can approximately be tracked by identifying the outline confidence areas that fall below one. However, an eye-blink detection via the outline confidence measure can only work if the algorithm’s detection rate is robust; i.e., the pupil is detected in more than 90% of valid eye image cases. We implemented the proposed two-step pre-processing method in MATLAB. The script is available on the GitHub repository of the *PupilEXT* project. Additionally, the recorded eye images are made available online together with the stereo calibration file. The data can directly be loaded into *PupilEXT* for a hands-on experience.

### Comparison of the Pupil Detection Approaches

A majority of pupil detection algorithms was evaluated based on their accuracy in estimating the pupil center (Table 1), as they are mainly intended for eye-tracking applications. One of the works evaluating the pupil fit was Świrski et al. (2012) in which their approach was compared against the Starburst algorithm. The pupil fit was assessed utilizing hand-labeled pupil measurements and the Hausdorff distance. The Hausdorff distance (Rote, 1991) thereby describes the maximum Euclidean distance of one ellipse to any point on the other ellipse (Świrski et al., 2012). Results show that the *Swirski* algorithm improves the detection rate for a five-pixel error threshold from 15% for Starburst to 87%, showing that not every eye-tracking algorithm is suited for pupil measurements. Fuhl et al. (2015) evaluated the *ExCuSe* algorithm, comparing their approach with the *Swirski* and *Starburst* algorithms. However, only the distance between the pupil center estimation and ground-truth was evaluated. The evaluation was performed on 18 datasets of pupil images captured under highly challenging real-world conditions. The detection rate for a five-pixel error threshold shows an average rate of 17% for *Starburst*, 40% for *Swirski* and 63% for *ExCuSe*.

A similar evaluation was repeated in the works of *ElSe* (Fuhl et al., 2016a), *PuRe* (Santini et al., 2018a), and *PuReST* (Santini et al., 2018b), where they conducted evaluations using overlapping datasets and the pupil center distance as a performance value. Within a five-pixel error threshold, the algorithm of Starburst shows a detection rate of 13.44, 28 to 36% for *Swirski*, 50 to 58% for *ExCuSe*, 66 to 69% for *ElSe*, 72% for *PuRe* and 87% for *PuReST*. In these evaluations, a performance loss for highly challenging recorded images was observed. Specifically, images with low-intensity contrast and pupils containing small reflections impaired the pupil detection algorithms. Santini et al. showed that the average runtime of the *PuReST* algorithm is 1.88 ms, compared with *PuRe* with 5.17 ms (Santini et al., 2018b), making *PuReST* the fastest approach with the highest pupil center detection rate. Note that these results apply to images that do not occur under laboratory conditions. Topal et al. (2017) evaluated the *APPD* algorithm (Topal et al., 2017) together with *Starburst*, *ElSe* and *Swirski*. The pupil fit and processing time were used to quantify the performance of the algorithms. For the pupil fit, the pupil localization was used, which quantifies the overlap ratio between the detected ellipse and the ground-truth, stated as [0, 1]. The results indicate a high pupil localization of 0.97 for *APPD* compared with 0.93 for *Swirski*, 0.92 for *ElSe* and 0.77 for Starburst. Additionally, Topal et al. measured an average computation time of 5.37 ms for *APPD*, 7.12 ms for *Else* (7 ms), 47.17 ms for *Swirski* (3.77 ms) and 49.22 ms for *Starburst* (100 ms). The numbers in parentheses define the originally reported runtime of the respective algorithms.

Based on the literature, it can be stated that *PuReST* is the top performer when evaluating the pupil’s center detection rate with highly noisy images. However, these results represent the detection rate with a five-pixel error threshold and do not state the accuracy of their pupil size measurements. Only the evaluation of Topal et al. (2017), Świrski et al. (2012) carried out a performance test on the pupil fit. Their results state a different picture, with *Swirski* performing better than *ElSe*.

Another aspect that could significantly affect the performance of a pupil detection algorithm is the parameters’ count. Each algorithm has a set of parameters that need to be tuned by the user to match the image composition. Selecting appropriate values may constitute a challenge for the user. Thus, the fewer parameters an algorithm possesses, the simpler its application. Comparing the number of parameters of the pupil detection algorithms, *Swirski* includes 11, followed by *Starburst* with five and *PuRe* and *PuReST* with three. *Else* and *ExCuSe* have only two parameters. We have stored in our proposed software *PupilEXT* the standard values of the algorithms as stated by the authors and additionally optimized three sets of parameters for pupil measurement applications under different image compositions.

### Validation of the Pupil Detection Algorithms

**FIGURE 9:**
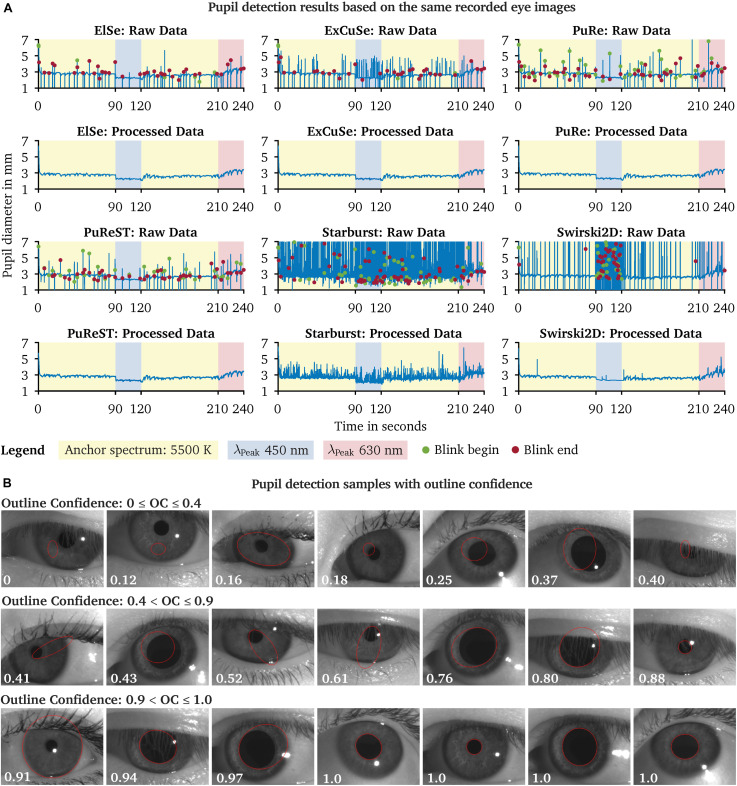
Comparison of the pupil detection algorithms based on the same eye image set and visualization of the pupil ellipse fit as a function of the outline confidence. **(A)** Eye images from one subject were recorded during a chromatic pupillometry experiment using *PupilEXT*. The pupil was exposed to LED spectra of the peak wavelengths 450 nm (L = 100.4 cd/m^2^ ± SD 0.23) and 630 nm (L = 101 cd/m^2^ ± SD 0.31) for 30 s. An anchor spectrum with a correlated color temperature (CCT) of 5,500 K (L = 201 cd/m^2^ ± SD 0.48) was turned on for 90 s between each stimulus. The pupil diameter from the recorded images was extracted using the available algorithms in *PupilEXT* and pre-processed to illustrate the algorithms’ detection differences. **(B)** For each detected diameter, an outline confidence measure is provided and used as an indicator to filter unreliable pupil fits from the dataset. Pupil fits from different measurement sessions are illustrated as a function of the outline confidence. We recommend discarding all pupil diameters with a lower outline confidence measure of 1.

We evaluated the captured eye images from our pupil experiment using the six available pupil detection algorithms in *PupilEXT*. Ideally, the pupil diameter should remain steady across the detection algorithms, as the same eye image sets were used for evaluation. However, due to the algorithms’ different parameters settings and approaches, the measured diameter may differ. In Figure 9A, we have plotted the detected raw pupil diameter from one experimental trial (240 s) to illustrate how differently the algorithms perform based on the same acquired image set. For each raw data plot panel, the respective pre-processed pupil data are illustrated, which were obtained using the proposed two-step method. The *ElSe*, *ExCuSe*, *PuRe* and *PuReST* algorithms achieved an acceptable pupil detection rate, visually noticeable through the lower number of artifacts in the respective raw dataset (Figure 9A). As discussed, the artifacts in the raw data can be filtered by removing the detected pupil diameter with an outline confidence of less than 1. In Figure 9B, we illustrated a sample of recorded pupil images with the respective outline confidence, showing that an invalid pupil fit can be detected and removed when using such a metric.

The *Starburst* algorithm caused a higher number of artifacts. Subsequent pre-processing of the raw data using the two-step method was not helpful, as the *Starburst* algorithm caused too many false detections. The *Swirski* algorithm had difficulties in detecting small pupil diameter at the 450-nm stimulus. After the invalid pupil data were filtered from the 450-nm time frame, there were almost no valid data left for linearly interpolating the missing values. Also, the *Swirski* algorithm had no robust detection rate for the pupil recording with the 630-nm spectrum. However, the cameras’ lenses were equipped with optical IR-high-pass filters so that the spectral-dependent detection quality was not due to the type of light spectrum. Each pupil detection algorithm has a certain number of parameters that need to be adjusted depending on the image resolution or how large the pupil is in relation to the image size. An incorrect combination of parameters could affect the pupil detection at differently sized diameters, as the algorithm itself could rule out smaller pupils.

The proposed technique for detecting eye blinks based on an outline confidence (Figure 8B) is highly affected by the detection rate. For example, it is no longer possible to distinguish between a false pupil fit or a closed eyelid at a higher rate of pupil detection artifacts (Figure 9A). Additionally, the *ExCuSe* algorithm offers a threshold value that can be used to detect eye blinks. In this way, values that indicate a closed eyelid will automatically be removed by the respective pupil detection algorithm itself, leading to the fact that a subsequent analysis of eye blinks is no longer possible. Therefore, an eye-blink recognition using the outline confidence seems to work well only with *PuRe* and *PuReST*.

**FIGURE 10:**
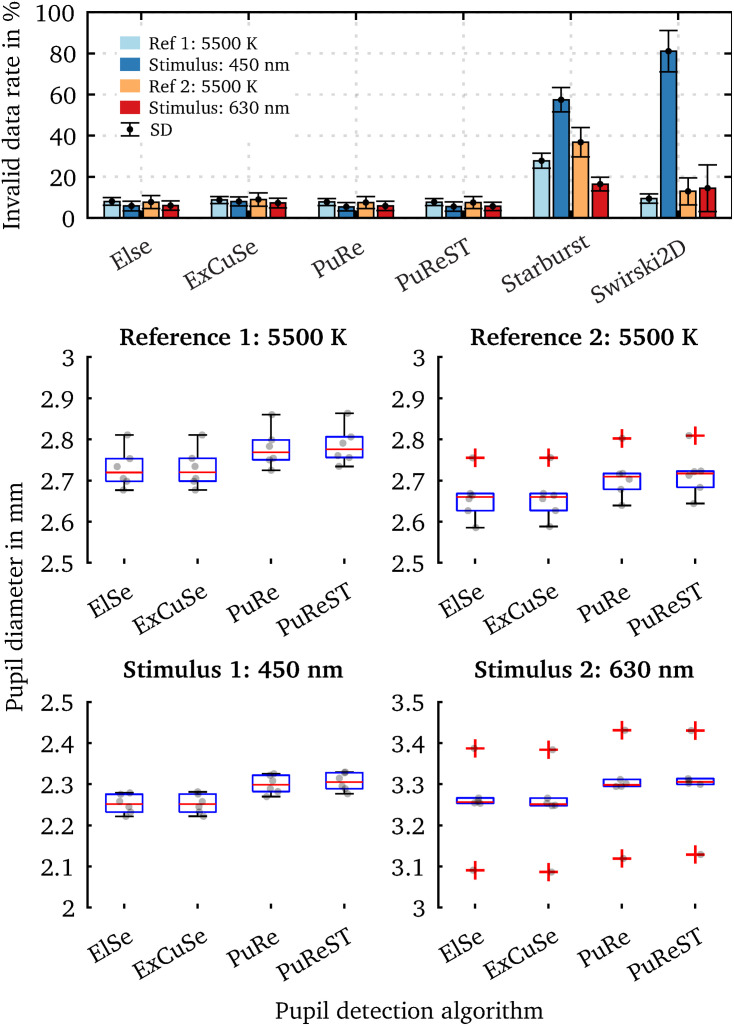
Percentage of invalid pupil fits inside the raw data, and the averaged steady-state pupil diameter of the last 5 s. Pupil data are from one subject with six repetitions in each condition. **(A)** Mean of the invalid data point count (outline confidence < 1) in per cent for each algorithm and used spectrum. A higher invalid data rate indicates that more raw data need to be removed due to inaccurate pupil fits. The *Starburst* algorithm did not provide an adequate detection rate, mainly observed at 450 nm (57.46% ± SD 5.93%) and the second reference spectrum (36.82% ± SD 7.1%). The *Swirski* algorithm showed a significant invalid detection rate of 81.09% ± SD 10.10% at 450 nm. Since the cameras were equipped with an IR-high-pass filter, the spectral-dependent pupil detection rate is mainly due to the differently sized pupil diameter. Due to the increased number of parameters from *Starburst* and *Swirski*, a generalization for small and large pupil diameters is more challenging. **(B)** The temporal pupil diameter was averaged over the last 5 s to compare how differently the pupil algorithms evaluate the same dataset. The *ElSe* and *ExCuSe* algorithms have approximately the same pupil diameter in all scenarios, which is due to the computation method’s similarities. The same applies to the *PuRe* and *PuReST* algorithms. The measured pupil diameter’s uncertainty range is on average 0.05 mm ± SD 0.004 mm, originating from the different detection results with the same dataset.

In Figure 10, we calculated the average percentage of the invalid data rate for each algorithm and spectrum separately to illustrate the pupil detection algorithms’ performance across the conducted pupil measurement trials. The invalid data rate is defined as the number of diameter values that had to be removed from the raw dataset when using the two-step pre-processing approach (Figure 8B). The *ElSe*, *ExCuSe*, *PuRe* and *PuReST* algorithms had a lower invalid data rate of 10%, indicating good detection performance across all measurement trials (Figure 10). The *Starburst* algorithm failed to perform a valid pupil fit at 450 nm in 58.46% SD 5.93% of cases. At the second reference spectrum (5,500 K), the pupil detections from *Starburst* failed in 36.82% SD 7.1% of the cases. Since the invalid pupil detection rate was higher than 10% for every stimulus spectrum, we assume that the performance of *Starburst* is independent of the parameter settings; possibly, the false pupil fits arise due to the contrast or resolution in the eye image. The *Swirski* algorithm’s performance suffered mainly at the 450-nm stimulus with an average error rate of 81.09% SD 10.10%. This behavior seems to be due to the algorithm’s parameters adjustments, as the invalid data rate is higher for smaller pupil diameters. Our results are in line with previous benchmarks from the literature, which showed that the *Starburst* and *Swirski* algorithms had lower detection rates (Fuhl et al., 2015, 2016a; Santini et al., 2018a, b). Note that the *Swirski* algorithm could have a better pupil fit, as it does not downscale the eye images before processing (Świrski et al., 2012; Topal et al., 2017). Since the *Swirski* algorithm has 11 free parameters that need to be adjusted, it is not a practical algorithm in our view because the detection method could suffer its robustness when using the wrong settings. The advantage of the pupil algorithms *ElSe*, *ExCuSe*, *PuRe* and *PuReST* is the smaller number of parameters that need to be set, leading to less error-proneness and practicability in conducting pupil measurements.

To better estimate how much the pupil diameter deviates depending on the used pupil algorithm, we evaluated the acquired eye images with the top-performing algorithms (*ElSe*, *ExCuSe*, *PuRe* and *PuReST*) and calculated the steady-state equilibrium pupil diameter. For this, we calculated the pupil diameter’s mean value over the last 5 s of a measurement. Figure 10B shows the steady-state pupil diameters from the six measurement trials. The scatter within a pupil algorithm is due to the pupil diameter’s intrasubject variability, which is mainly induced by cognitive effects and can be up to 0.5 mm (Zandi et al., 2020; Zandi and Khanh, 2021). The absolute mean pupil diameter differences between the *ElSe* and *ExCuSe* algorithms are negligible with 6 ⋅ 10^–4^ mm at 450 nm and 0.0041 mm at 630 nm, which are due to the same detection approaches. The same was applied for the *PuRe* and *PuReST* algorithms with an absolute mean diameter difference of 0.0061 mm at 450 nm and 0.0051 mm at 630 nm. The *PuReST* algorithm was an extension of *PuRe*, allowing faster pupil detections and explaining the similar pupil fits. However, the mean difference between the algorithm groups *ElSe*/*ExCuse* and *PuRe*/*PuReST* is 0.054 mm SD 0.0043 mm. This is particularly interesting because it indicates how much the measured pupil diameter can deviate when different detection method approaches are applied to the same eye image set. Therefore, in cognitive studies in which the pupil diameters’ mean difference is less than 0.1 mm, we highly recommend reporting the algorithm and respective parameter settings. The parameters that we used for our pupil detection experiments are stored in the *PupilEXT* software and also available on the GitHub repository of this project.

### Determining the Pupil Measurement Accuracy

**FIGURE 11:**
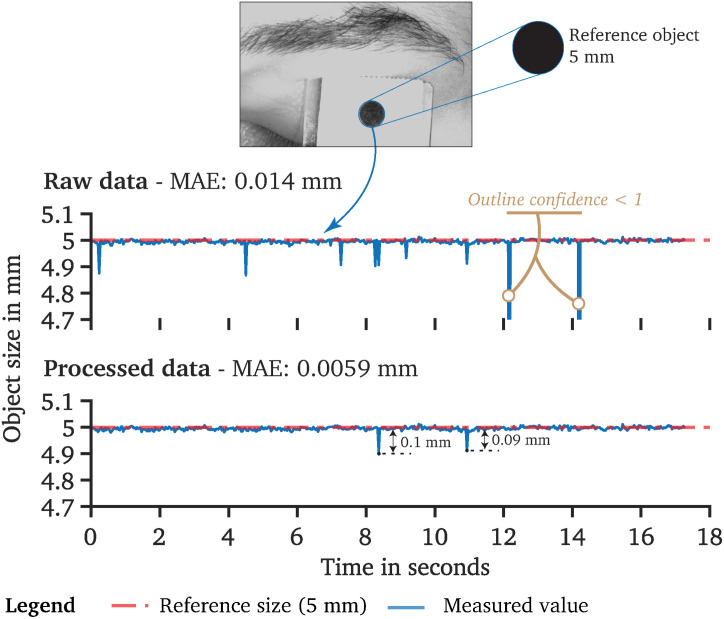
Determination of the used stereo camera system’s measurement accuracy. A reference object of known size (5 mm) was placed in front of the observers’ occluded eye. The diameter of the object was tracked using the *PuRe* algorithm. Based on the raw data, a mean absolute error (MAE) of 0.014 mm can be determined. However, the raw data contain artifacts that we have removed using the proposed two-step pre-processing approach. After pre-processing, we can state a measurement accuracy of 0.0059 mm (MAE).

The accuracy of the pupil measurement can by characterized with *PupilEXT* by two approaches. First, the validation process of the stereo calibration determines the quality of the system, indicated by the reprojection error in MAE within *PupilEXT*. However, such a metric does not include the inaccuracies caused by pupil detection methods. Therefore, it is advisable for checking the validity of the system by a circular formed reference object. For this, we placed a reference object of known size (5 mm) in front of the subject’s eye and determined the diameter using a pupil detection algorithm in *PupilEXT* (Figure 11).

The measured raw data of the reference object showed a MAE of 0.014 mm. After pre-processing the data with the two-step method, a MAE accuracy of 0.0059 mm was achieved with our prototyped system. It should be noted that such a measurement accuracy is still an idealized approximation since the reference object was kept still without interference. After pre-processing, isolated peaks remained with an amplitude of 0.1 mm. However, remaining pupil data are usually smoothed, making such remaining isolated peaks negligible.

### Limitations of the Proposed Pupillometry Toolbox

The current version of *PupilEXT* offers a comprehensive solution for pupillometry. However, the software is not designed for two-eye measurements, as only one eye at the same time can be captured. We recommend positioning the ROI in the live view of *PupilEXT* software over one eye to let the algorithms iterate inside the specified region if two eyes are visible in the image. Furthermore, an online pupil measurement can only be carried out with Basler branded cameras. In the future, the integration of other camera brands is possible through the implemented camera class. However, externally acquired images from other camera brands can be loaded into *PupilEXT* for offline pupil detections, making it possible to use the software even without purchasing a Basler camera.

Currently, the implemented pupil algorithms perform their computations on the CPU. Therefore, we recommend using the *PuRe* or *PuReST* algorithm for real-time pupil measurements with a higher frame rate between 60 and 120 fps, as the detection approaches shine with low processing times. In the future, it would be desirable to perform calculations directly on a graphics processing unit (GPU) during an online measurement, making higher frame rates for all integrated pupil detection methods possible. Note that we did not implement a limiting threshold of the frame rate level inside the *PupilEXT* software. The frame rate is limited by the respective pupil detection algorithm’s processing time, which can vary depending on the used computer. If the frame rate is too high for the computer during an online pupil measurement, the images will be stored in the machine’s memory buffer and fed to the pupil algorithm one by one. In such cases, there is the risk that the working memory will overflow when operating *PupilEXT* for longer times in such a mode. Therefore, the camera fps should ideally be on the same level as the processing fps. Both metrics are stated in the live view panel of *PupilEXT*. Note that on our computer (Intel Core i7-9700K), we performed pupil measurements in stereo mode at 120 Hz without any issues when using the *PuReST* or *PuRe* algorithm. Even higher frame rates are possible in the single-camera mode because only the image from one camera has to be processed. Alternatively, eye images can be captured on the disk for later pupil detection, allowing higher frame rates. This function is available for both mono and stereo camera modes.

## Discussion

The idea of replacing commercial systems with open-source solutions is currently pushed by working groups topically working on eye-tracking devices (Santini et al., 2017; Arvin et al., 2020). The advantage of eye-tracking research is that standardized metrics exist that reflect the accuracy of a detected gaze point (Holmqvist et al., 2012). In pupillometry research, metrics on the pupil fit’s measurement accuracy is usually not stated, mainly because most applied systems do not allow manual verification after conducted experiments. The lack of missing pupil fit metrics in commercial eye-tracking systems applied for pupil measurement motivated recent works, attempting to develop procedures or provide at least pupil measurement error information of widely used systems (Klingner, 2010; Gagl et al., 2011; Brisson et al., 2013; Hayes and Petrov, 2016; Murray et al., 2017; Wang et al., 2017; Titz et al., 2018; Coyne et al., 2019). Mathematically, the pupil center’s accuracy detection is just an indicator for a good pupil fit but does not ensure it. For example, the pupil center can be correct for cases in which the gaze point differs from the camera’s optical axis (eye rotation), but the detected pupil diameter can be estimated incorrectly due to the perspective distortion of the pupil image (pupil foreshortening error) (Hayes and Petrov, 2016). Additionally, it is not directly possible to reproduce the pupil fit’s accuracy from the pupil center accuracy, which is mainly stated in the datasheet of eye-tracking devices. Suppose studies indicate an effect on the pupil diameter of 0.5 mm. In that case, ideally, there should be a procedure to verify that both the camera system and the applied pupil detection method can detect such small diameter margins. For example, the recently published work “Standards in Pupillography” (Kelbsch et al., 2019) rarely paid attention to possible technical- and software-induced measurement errors, although this could highly affect the validity and conclusions of research results. By comparing the pupil detection algorithms, we showed that a measurement error of up to 0.05 mm could occur with identical eye images, induced solely by the type of used detection algorithm itself. In commercial systems where it is usually unknown which pupil detection algorithm is applied, comparisons between study results in such a measurement range are difficult. Therefore, the camera’s spatial resolution specification or the pupil center’s measurement accuracy is insufficient for pupil measurements. From our perspective, a uniform measurement platform is essential for pupillometry, ensuring comparability and reproducibility. By verifying our proposed *PupilEXT* set up with a reference object, we offer the possibility to test and state the accuracy of the pupil’s fit directly. Furthermore, the proposed system ensures reproducing pupil examinations results by using the captured images in the offline analysis mode of *PupilEXT*.

With *PupilEXT*, we have developed the first freely accessible integrated end-to-end pupil measurement platform consisting of hardware and software for professional pupillometry research in vision science. Pupil measurement can be carried out in a one- or two-camera mode. The calibration and validation procedure in *PupilEXT* are intended to provide a transparent way in reporting the measurement accuracy of a conducted pupil study. The specification of measurement accuracies is currently a major issue in pupil research since only in few publications is the validity of the pupil tracking’s accuracy stated. This is mainly due to the use of commercial systems that usually do not support validation procedures of pupil measurement pipelines. The complete software, embedded code and printed circuit board (PCB) layout of the NIR illumination are provided as an open-source project. We provide three Supplementary Videos to illustrate the handling of *PupilEXT*. The instruction, details about the installation and video tutorials can be found at the project’s website (see text footnote 1).

As a next step, it is planned to add a gaze calibration routine to *PupilEXT* to support eye-tracking applications. Currently, we only support Basler branded cameras, but it is possible to add additional industrial camera types into *PupilEXT* since the camera access is separated from the core of the proposed software. The feature of determining the pupil diameter from externally captured images could perhaps make *PupilEXT* a standardized measurement suitable for pupil research. For this aim, we will investigate in the next studies the tracking accuracy of the integrated pupil algorithms with ground-truth images, leading to a better estimation of real-world inaccuracies under laboratory conditions.

## Data Availability Statement

The software PupilEXT as well the embedded program of the microcontroller, and PCB layout of the NIR illumination are available on the following GitHub page: https://github.com/openPupil/Open-PupilEXT.

## Ethics Statement

The studies involving human participants were reviewed and approved by Technical University of Darmstadt. The patients/participants provided their written informed consent to participate in this study. Written informed consent was obtained from the individual(s) for the publication of any potentially identifiable images or data included in this article.

## Author Contributions

BZ had the initial idea, supervised the project, and designed the first concept of the pupillometry platform. ML was the core software developer with contributions and supervision of BZ. BZ wrote the original draft of the manuscript with contributions of ML. ML provided the literature research and summary of existing pupil detection algorithms under the supervision of BZ. BZ created the figures with contributions of ML and AH. BZ developed and programmed the hardware trigger concept. GS, AH, TK, ML, and BZ performed review and editing of the original draft. GS, AH, TK, ML, and BZ developed the testing methodology. ML and BZ performed testing. All authors read and agreed to the submitted version of the manuscript.

## Conflict of Interest

The authors declare that the research was conducted in the absence of any commercial or financial relationships that could be construed as a potential conflict of interest.

## Footnotes

1 https://github.com/openPupil/Open-PupilEXT

## References

[B1] Allen A. E. Martial F. P. Lucas R. J. (2019). Form vision from melanopsin in humans. *Nat. Commun.* 10 1–10. 10.1038/s41467-019-10113-3 31118424 PMC6531428

[B2] Aminihajibashi S. Hagen T. Andreassen O. A. Laeng B. Espeseth T. (2020). The effects of cognitive abilities and task demands on tonic and phasic pupil sizes. *Biol. Psychol.* 156:107945. 10.1016/j.biopsycho.2020.107945 32889001

[B3] Arvin S. Rasmussen R. Yonehara K. (2020). EyeLoop: an open-source, high-speed eye-tracker designed for dynamic experiments. *bioRxiv* [Preprint]. 10.1101/2020.07.03.186387

[B4] Attard-Johnson J. Ciardha C. Ó Bindemann M. (2019). Comparing methods for the analysis of pupillary response. *Behav. Res. Methods* 51 83–95. 10.3758/s13428-018-1108-6 30324564 PMC6420434

[B5] Barrionuevo P. A. McAnany J. J. Zele A. J. Cao D. (2018). Non-linearities in the rod and cone photoreceptor inputs to the afferent pupil light response. *Front. Neurol.* 9:1140. 10.3389/fneur.2018.01140 30622511 PMC6308191

[B6] Barten P. G. (1999). “Contrast sensitivity of the human eye and its effects on image quality,” in *Proceedings of the Contrast Sensit. Hum. Eye Its Eff. Image Qual*, 27–29. 10.1117/3.353254

[B7] Beatty J. (1982). Task-evoked pupillary responses, processing load, and the structure of processing resources. *Psychol. Bull.* 91 276–292. 10.1037/0033-2909.91.2.276 7071262

[B8] Beatty J. Wagoner B. L. (1978). Pupillometric signs of brain activation vary with level of cognitive processing. *Science* 199 1216–1218. 10.1126/science.628837 628837

[B9] Berman S. M. Jewett D. L. Fein G. Saika G. Ashford F. (1990). Photopic luminance does not always predict perceived room brightness. *Light. Res. Technol.* 22 37–41. 10.1177/096032719002200103

[B10] Berson D. M. (2003). Strange vision: ganglion cells as circadian photoreceptors. *Trends Neurosci.* 26 314–320. 10.1016/S0166-2236(03)00130-9 12798601

[B11] Berson D. M. Dunn F. A. Takao M. (2002). Phototransduction by retinal ganglion cells that set the circadian clock. *Science* 295 1070–1073. 10.1126/science.1067262 11834835

[B12] Besenecker U. C. Bullough J. D. (2017). Investigating visual mechanisms underlying scene brightness. *Light. Res. Technol.* 49 16–32. 10.1177/1477153516628168

[B13] Binda P. Gamlin P. D. (2017). Renewed attention on the pupil light reflex. *Trends Neurosci.* 40 455–457. 10.1016/j.tins.2017.06.007 28693846 PMC5562352

[B14] Blackie C. A. Howland H. C. (1999). An extension of an accommodation and convergence model of emmetropization to include the effects of illumination intensity. *Ophthalmic Physiol. Opt.* 19 112–125. 10.1016/S0275-5408(98)00077-5 10615447

[B15] Bodmann H. W. (1992). Elements of photometry, brightness and visibility. *Light. Res. Technol.* 24 29–42. 10.1177/096032719202400104

[B16] Bombeke K. Duthoo W. Mueller S. C. Hopf J. M. Boehler C. N. (2016). Pupil size directly modulates the feedforward response in human primary visual cortex independently of attention. *Neuroimage* 127 67–73. 10.1016/j.neuroimage.2015.11.072 26658931

[B17] Bonmati-Carrion M. A. Hild K. Isherwood C. Sweeney S. J. Revell V. L. Skene D. J. (2016). Relationship between human pupillary light reflex and circadian system status. *PLoS One* 11:e0162476. 10.1371/journal.pone.0162476 27636197 PMC5026360

[B18] Brainard G. C. Hanifin J. R. Greeson J. M. Byrne B. Glickman G. Gerner E. (2001). Action spectrum for melatonin regulation in humans: evidence for a novel circadian photoreceptor. *J. Neurosci.* 21 6405–6412. 10.1523/jneurosci.21-16-06405.2001 11487664 PMC6763155

[B19] Brisson J. Mainville M. Mailloux D. Beaulieu C. Serres J. Sirois S. (2013). Pupil diameter measurement errors as a function of gaze direction in corneal reflection eyetrackers. *Behav. Res. Methods* 45 1322–1331. 10.3758/s13428-013-0327-0 23468182

[B20] Campbell F. W. (1957). The depth of field of the human eye. *Opt. Acta Int. J. Opt.* 4 157–164. 10.1080/713826091

[B21] Campbell F. W. Gubisch R. W. (1966). Optical quality of the human eye. *J. Physiol.* 186 558–578. 10.1113/jphysiol.1966.sp008056 5972153 PMC1395916

[B22] Canver M. C. Canver A. C. Revere K. E. Amado D. Bennett J. Chung D. C. (2014). Novel mathematical algorithm for pupillometric data analysis. *Comput. Methods Programs Biomed.* 113 221–225. 10.1016/j.cmpb.2013.08.008 24129048

[B23] Carle C. F. James A. C. Rosli Y. Maddess T. (2019). Localization of neuronal gain control in the pupillary response. *Front. Neurol.* 10:203. 10.3389/fneur.2019.00203 30930833 PMC6423807

[B24] Chen S. Epps J. (2014). Efficient and robust pupil size and blink estimation from near-field video sequences for human-machine interaction. *IEEE Trans. Cybern.* 44 2356–2367. 10.1109/TCYB.2014.2306916 24691198

[B25] Cherng Y.-G. Baird T. Chen J.-T. Wang C.-A. (2020). Background luminance effects on pupil size associated with emotion and saccade preparation. *Sci. Rep.* 10:15718. 10.1038/s41598-020-72954-z 32973283 PMC7515892

[B26] Chougule P. S. Najjar R. P. Finkelstein M. T. Kandiah N. Milea D. (2019). Light-induced pupillary responses in Alzheimer’s disease. *Front. Neurol.* 10:360. 10.3389/fneur.2019.00360 31031692 PMC6473037

[B27] CIE (2011). *CIE:200:2001: Supplementary System of Photometry.* Available online at: http://cie.co.at/publications/cie-supplementary-system-photometry (accessed July 23, 2020).

[B28] Clewett D. Gasser C. Davachi L. (2020). Pupil-linked arousal signals track the temporal organization of events in memory. *Nat. Commun.* 11:4007. 10.1038/s41467-020-17851-9 32782282 PMC7421896

[B29] Connelly M. A. Brown J. T. Kearns G. L. Anderson R. A. St Peter S. D. Neville K. A. (2014). Pupillometry: a non-invasive technique for pain assessment in paediatric patients. *Arch. Dis. Child.* 99 1125–1131. 10.1136/archdischild-2014-306286 25187497 PMC4395877

[B30] Coyne J. T. Brown N. Foroughi C. K. Sibley C. M. (2019). Improving pupil diameter measurement accuracy in a remote eye tracking system. *Proc. Hum. Factors Ergon. Soc. Annu. Meet.* 63 49–53. 10.1177/1071181319631176

[B31] Crawford B. H. (1936). The dependence of pupil size upon external light stimulus under static and variable conditions. *Proc. R. Soc. London. Ser. B Biol. Sci.* 121 376–395. 10.1098/rspb.1936.0072

[B32] Crippa S. V. Domellöf F. P. Kawasaki A. (2018). Chromatic pupillometry in children. *Front. Neurol.* 9:669. 10.3389/fneur.2018.00669 30174642 PMC6107754

[B33] de Groot S. G. Gebhard J. W. (1952). Pupil size as determined by adapting luminance. *J. Opt. Soc. Am.* 42:492. 10.1364/JOSA.42.000492 14939111

[B34] de Winter J. C. F. Petermeijer S. M. Kooijman L. Dodou D. (2021). Replicating five pupillometry studies of Eckhard Hess. *Int. J. Psychophysiol* 165 145–205. 10.1016/j.ijpsycho.2021.03.003 33766646

[B35] Dey S. Samanta D. (2007). “An efficient approach for pupil detection in iris images,” in *Proceedings of the 15th Int. Conf. Adv. Comput. Commun. ADCOM 2007*, Guwahati, 382–387. 10.1109/adcom.2007.79

[B36] Do M. T. H. (2019). Melanopsin and the intrinsically photosensitive retinal ganglion cells: biophysics to behavior. *Neuron* 104 205–226. 10.1016/j.neuron.2019.07.016 31647894 PMC6944442

[B37] Do M. T. H. Kang S. H. Xue T. Zhong H. Liao H. W. Bergles D. E. (2009). Photon capture and signalling by melanopsin retinal ganglion cells. *Nature* 457 281–287. 10.1038/nature07682 19118382 PMC2794210

[B38] Ebisawa Y. (1994). “Improved video-based eye-gaze detection method,” in *Proceedings of the Conf. Proc. - 10th Anniv. IMTC 1994 Adv. Technol. I M. 1994 IEEE Instrum. Meas. Technol. Conf*, Hamamatsu, 963–966. 10.1109/IMTC.1994.351964

[B39] Ebisawa Y. (2004). “Realtime 3D position detection of human pupil,” in *Proceedings of the 2004 IEEE Symp. Virtual Environ. Human-Computer Interfaces Meas. Syst. VECIMS*, Boston, MA, 8–12. 10.1109/vecims.2004.1397176

[B40] Ecker J. L. Dumitrescu O. N. Wong K. Y. Alam N. M. Chen S.-K. LeGates T. (2010). Melanopsin-expressing retinal ganglion-cell photoreceptors: cellular diversity and role in pattern vision. *Neuron* 67 49–60. 10.1016/j.neuron.2010.05.023 20624591 PMC2904318

[B41] Eivazi S. Santini T. Keshavarzi A. Kübler T. Mazzei A. (2019). “Improving real-time CNN-based pupil detection through domain-specific data augmentation,” in *Proceedings of the Eye Tracking Research and Applications Symposium (ETRA)*, (New York, NY: Association for Computing Machinery), 1–6. 10.1145/3314111.3319914

[B42] Freedman M. S. (1999). Regulation of mammalian circadian behavior by non-rod, non-cone, ocular photoreceptors. *Science* 284 502–504. 10.1126/science.284.5413.502 10205061

[B43] Fuhl W. Eivazi S. Hosp B. Eivazi A. Rosenstiel W. Kasneci E. (2018a). “BORE: boosted-oriented edge optimization for robust, real time remote pupil center detection,” in *Proceedings of the Eye Tracking Research and Applications Symposium (ETRA)*, (New York, NY: Association for Computing Machinery), 1–5. 10.1145/3204493.3204558

[B44] Fuhl W. Geisler D. Santini T. Appel T. Rosenstiel W. Kasneci E. (2018b). “CBF: circular binary features for robust and real-time pupil center detection,” in *Proceedings of the Eye Tracking Research and Applications Symposium (ETRA)*, (New York, NY: Association for Computing Machinery), 1–6. 10.1145/3204493.3204559

[B45] Fuhl W. Kübler T. Sippel K. Rosenstiel W. Kasneci E. (2015). “ExCuSe: robust pupil detection in real-world scenarios,” in *Computer Analysis of Images and Patterns*, eds Azzopardi G. Petkov N. (Cham: Springer), 39–51. 10.1007/978-3-319-23192-1_4

[B46] Fuhl W. Santini T. Kasneci G. Kasneci E. (2016b). *PupilNet: Convolutional Neural Networks for Robust Pupil Detection.* Available online at: http://arxiv.org/abs/1601.04902 (accessed October 6, 2020).

[B47] Fuhl W. Santini T. Kasneci G. Rosenstiel W. Kasneci E. (2017). *PupilNet v2.0: Convolutional Neural Networks for CPU based real time Robust Pupil Detection.* Available online at: http://arxiv.org/abs/1711.00112 (accessed October 6, 2020).

[B48] Fuhl W. Santini T. C. Kübler T. Kasneci E. (2016a). ElSe: ellipse selection for robust pupil detection in real-world environments. *Eye Track. Res. Appl. Symp.* 14 123–130. 10.1145/2857491.2857505

[B49] Gagl B. Hawelka S. Hutzler F. (2011). Systematic influence of gaze position on pupil size measurement: analysis and correction. *Behav. Res. Methods* 43 1171–1181. 10.3758/s13428-011-0109-5 21637943 PMC3218283

[B50] Gamlin P. D. R. McDougal D. H. Pokorny J. Smith V. C. Yau K.-W. Dacey D. M. (2007). Human and macaque pupil responses driven by melanopsin-containing retinal ganglion cells. *Vision Res.* 47 946–954. 10.1016/j.visres.2006.12.015 17320141 PMC1945238

[B51] Goñi S. Echeto J. Villanueva A. Cabeza R. (2004). Robust algorithm for pupil-glint vector detection in a video-oculography eyetracking system. *Proc. Int. Conf. Pattern Recognit.* 4 941–944. 10.1109/ICPR.2004.1333928

[B52] Gooley J. J. Lu J. Chou T. C. Scammell T. E. Saper C. B. (2001). Melanopsin in cells of origin of the retinohypothalamic tract. *Nat. Neurosci.* 4:1165. 10.1038/nn768 11713469

[B53] Granholm E. L. Panizzon M. S. Elman J. A. Jak A. J. Hauger R. L. Bondi M. W. (2017). Pupillary responses as a biomarker of early risk for Alzheimer’s disease. *J. Alzheimer’s Dis.* 56 1419–1428. 10.3233/JAD-161078 28157098 PMC5808562

[B54] Güler A. D. Ecker J. L. Lall G. S. Haq S. Altimus C. M. Liao H. W. (2008). Melanopsin cells are the principal conduits for rod-cone input to non-image-forming vision. *Nature* 453 102–105. 10.1038/nature06829 18432195 PMC2871301

[B55] Hattar S. (2002). Melanopsin-containing retinal ganglion cells: architecture, projections, and intrinsic photosensitivity. *Science* 295 1065–1070. 10.1126/science.1069609 11834834 PMC2885915

[B56] Hattar S. Kumar M. Park A. Tong P. Tung J. Yau K.-W. (2006). Central projections of melanopsin-expressing retinal ganglion cells in the mouse. *J. Comp. Neurol.* 497 326–349. 10.1002/cne.20970 16736474 PMC2885916

[B57] Hattar S. Lucas R. J. Mrosovsky N. Thompson S. Douglas R. H. Hankins M. W. (2003). Melanopsin and rod—cone photoreceptive systems account for all major accessory visual functions in mice. *Nature* 424 76–81. 10.1038/nature01761 12808468 PMC2885907

[B58] Hayes T. R. Petrov A. A. (2016). Mapping and correcting the influence of gaze position on pupil size measurements. *Behav. Res. Methods* 48 510–527. 10.3758/s13428-015-0588-x 25953668 PMC4637269

[B59] Hermans S. Smet K. A. G. Hanselaer P. (2018). Brightness model for neutral self-luminous stimuli and backgrounds. *LEUKOS J. Illum. Eng. Soc. North Am.* 14 231–244. 10.1080/15502724.2018.1448280

[B60] Hiley J. B. Redekopp A. H. Fazel-Rezai R. (2006). A low cost human computer interface based on eye tracking. *Annu. Int. Conf. IEEE Eng. Med. Biol. Proc.* 2006 3226–3229. 10.1109/IEMBS.2006.260774 17946167

[B61] Holladay L. L. (1926). The fundamentals of glare and visibility. *J. Opt. Soc. Am.* 12:271. 10.1364/JOSA.12.000271

[B62] Holmqvist K. Nyström M. Mulvey F. (2012). “Eye tracker data quality: what it is and how to measure it,” in *Proceedings of the Symposium on Eye Tracking Research and Applications ETRA ‘12*, (New York, NY: ACM Press), 45. 10.1145/2168556.2168563

[B63] Hosp B. Eivazi S. Maurer M. Fuhl W. Geisler D. Kasneci E. (2020). RemoteEye: an open-source high-speed remote eye tracker: Implementation insights of a pupil- and glint-detection algorithm for high-speed remote eye tracking. *Behav. Res. Methods* 52 1387–1401. 10.3758/s13428-019-01305-2 32212086

[B64] Hreidarsson A. B. (1982). Pupil size in insulin-dependent diabetes. Relationship to duration, metabolic control, and long-term manifestations. *Diabetes* 31 442–448. 10.2337/diab.31.5.442 6759259

[B65] Hu X. Hisakata R. Kaneko H. (2019). Effects of spatial frequency and attention on pupillary response. *J. Opt. Soc. Am. A* 36:1699. 10.1364/josaa.36.001699 31674435

[B66] Hutchinson T. E. White K. P. Martin W. N. Reichert K. C. Frey L. A. (1989). Human-computer interaction using eye-gaze input. *IEEE Trans. Syst. Man Cybern.* 19 1527–1534. 10.1109/21.44068

[B67] Javadi A. H. Hakimi Z. Barati M. Walsh V. Tcheang L. (2015). Set: a pupil detection method using sinusoidal approximation. *Front. Neuroeng.* 8:4. 10.3389/fneng.2015.00004 25914641 PMC4391030

[B68] Jennings B. J. Martinovic J. (2014). Luminance and color inputs to mid-level and high-level vision. *J. Vis.* 14 1–17. 10.1167/14.2.9 24520151

[B69] Jepma M. Nieuwenhuis S. (2011). Pupil diameter predicts changes in the exploration-exploitation trade-off: evidence for the adaptive gain theory. *J. Cogn. Neurosci.* 23 1587–1596. 10.1162/jocn.2010.21548 20666595

[B70] Joshi S. (2021). Pupillometry: arousal state or state of mind? *Curr. Biol.* 31 R32–R34. 10.1016/j.cub.2020.11.001 33434486

[B71] Joyce D. S. Feigl B. Kerr G. Roeder L. Zele A. J. (2018). Melanopsin-mediated pupil function is impaired in Parkinson’s disease. *Sci. Rep.* 8:7796. 10.1038/s41598-018-26078-0 29773814 PMC5958070

[B72] Kassner M. Patera W. Bulling A. (2014). “Pupil: an open source platform for pervasive eye tracking and mobile gaze-based interaction,” in *Proceedings of the UbiComp 2014 - Adjun. Proc. 2014 ACM Int. Jt. Conf. Pervasive Ubiquitous Comput*, Seattle WA, 1151–1160. 10.1145/2638728.2641695

[B73] Keil A. Albuquerque G. Berger K. Magnor M. A. (2010). “Real-time gaze tracking with a consumer-grade video camera,” in *Proceedings of the 18th Int. Conf. Cent. Eur. Comput. Graph. Vis. Comput. Vision, WSCG 2010 - Co-operation with EUROGRAPHICS, Full Pap. Proc*, Plzen, Czech Republic. 129–134.

[B74] Kelbsch C. Strasser T. Chen Y. Feigl B. Gamlin P. D. Kardon R. (2019). Standards in pupillography. *Front. Neurol.* 10:129. 10.3389/fneur.2019.00129 30853933 PMC6395400

[B75] Kercher C. Azinfar L. Dinalankara D. M. R. Takahashi T. N. Miles J. H. Yao G. (2020). A longitudinal study of pupillary light reflex in 6- to 24-month children. *Sci. Rep.* 10:1205. 10.1038/s41598-020-58254-6 31988320 PMC6985190

[B76] Klingner J. (2010). “The pupillometric precision of a remote video eye tracker,” in *Proceedings of the 2010 Symposium on Eye-Tracking Research & Applications, ETRA 2010*, Austin, TX, 259–262. 10.1145/1743666.1743727

[B77] Kobashi H. Kamiya K. Ishikawa H. Goseki T. Shimizu K. (2012). Daytime variations in pupil size under photopic conditions. *Optom. Vis. Sci.* 89 197–202. 10.1097/OPX.0b013e31824048a9 22179219

[B78] Kret M. E. Sjak-Shie E. E. (2019). Preprocessing pupil size data: guidelines and code. *Behav. Res. Methods* 51 1336–1342. 10.3758/s13428-018-1075-y 29992408 PMC6538573

[B79] Kumar N. Kohlbecher S. Schneider E. (2009). “A novel approach to video-based pupil tracking,” in *Proceedings of the Conf. Proc. IEEE Int. Conf. Syst. Man Cybern*, San Antonio, TX, 1255–1262. 10.1109/ICSMC.2009.5345909

[B80] La Morgia C. Carelli V. Carbonelli M. (2018). Melanopsin retinal ganglion cells and pupil: clinical implications for neuro-ophthalmology. *Front. Neurol.* 9:1047. 10.3389/fneur.2018.01047 30581410 PMC6292931

[B81] Lanatà A. Armato A. Valenza G. Scilingo E. P. (2011). “Eye tracking and pupil size variation as response to affective stimuli: a preliminary study,” in *Proceedings of the 2011 5th International Conference on Pervasive Computing Technologies for Healthcare and Workshops, PervasiveHealth* Dublin, 78–84. 10.4108/icst.pervasivehealth.2011.246056

[B82] Lee J. W. Cho C. W. Shin K. Y. Lee E. C. Park K. R. (2012). 3D gaze tracking method using Purkinje images on eye optical model and pupil. *Opt. Lasers Eng.* 50 736–751. 10.1016/j.optlaseng.2011.12.001

[B83] Lemercier A. Guillot G. Courcoux P. Garrel C. Baccino T. Schlich P. (2014). Pupillometry of taste: methodological guide – from acquisition to data processing - and toolbox for MATLAB. *Quant. Methods Psychol.* 10 179–195. 10.20982/tqmp.10.2.p179

[B84] Lennie P. Pokorny J. Smith V. C. (1993). Luminance. *J. Opt. Soc. Am. A* 10:1283. 10.1364/JOSAA.10.001283 8320586

[B85] Li D. Winfield D. Parkhurst D. J. (2005). “Starburst: a hybrid algorithm for video-based eye tracking combining feature-based and model-based approaches,” in *Proceedings of the 2005 IEEE Computer Society Conference on Computer Vision and Pattern Recognition (CVPR’05) - Workshops*, (San Diego, CA: IEEE), 79. 10.1109/CVPR.2005.531

[B86] Li J. Li S. Chen T. Liu Y. (2018). A geometry-appearance-based pupil detection method for near-infrared head-mounted cameras. *IEEE Access* 6 23242–23252. 10.1109/ACCESS.2018.2828400

[B87] Lim J. K. H. Li Q. X. He Z. Vingrys A. J. Wong V. H. Y. Currier N. (2016). The eye as a biomarker for Alzheimer’s disease. *Front. Neurosci.* 10:536. 10.3389/fnins.2016.00536 27909396 PMC5112261

[B88] Lin L. Pan L. Wei L. F. Yu L. (2010). “A robust and accurate detection of pupil images,” in *Proceedings of the - 2010 3rd Int. Conf. Biomed. Eng. Informatics, BMEI 2010*, Yantai, 70–74. 10.1109/BMEI.2010.5639646

[B89] Lin X. Craig J. Dean S. Klette G. Klette R. (2003). *Accurately Measuring the Size of the Pupil of the Eye.* Auckland: CITR, The University of Auckland.

[B90] Long X. Tonguz O. K. Kiderman A. (2007). “A high speed eye tracking system with robust pupil center estimation algorithm,” in *Proceedings of the Annual International Conference of the IEEE Engineering in Medicine and Biology*, Lyon, 3331–3334. 10.1109/IEMBS.2007.4353043 18002709

[B91] Lucas R. J. Allen A. E. Milosavljevic N. Storchi R. Woelders T. (2020). Can We See with Melanopsin? *Annu. Rev. Vis. Sci.* 6 453–468. 10.1146/annurev-vision-030320-041239 32491960

[B92] Lucas R. J. Douglas R. H. Foster R. G. (2001). Characterization of an ocular photopigment capable of driving pupillary constriction in mice. *Nat. Neurosci.* 4 621–626. 10.1038/88443 11369943

[B93] Lucas R. J. Peirson S. N. Berson D. M. Brown T. M. Cooper H. M. Czeisler C. A. (2014). Measuring and using light in the melanopsin age. *Trends Neurosci.* 37 1–9. 10.1016/j.tins.2013.10.004 24287308 PMC4699304

[B94] Maclean H. Dhillon B. (1993). Pupil cycle time and human immunodeficiency virus (hiv) infection. *Eye* 7 785–786. 10.1038/eye.1993.184 8119434

[B95] Manuri F. Sanna A. Petrucci C. P. (2020). PDIF: pupil detection after isolation and fitting. *IEEE Access* 8 30826–30837. 10.1109/ACCESS.2020.2973005

[B96] Martinikorena I. Cabeza R. Villanueva A. Urtasun I. Larumbe A. (2018). Fast and robust ellipse detection algorithm for head-mounted eye tracking systems. *Mach. Vis. Appl.* 29 845–860. 10.1007/s00138-018-0940-0

[B97] Mazziotti R. Carrara F. Viglione A. Lupori L. Lo Verde L. Benedetto A. (2021). MEYE: web-app for translational and real-time pupillometry. *bioRxiv* [Preprint]. 10.1101/2021.03.09.434438 PMC8489024 34518364

[B98] Merritt S. L. Schnyders H. C. Patel M. Basner R. C. O’Neill W. (2004). Pupil staging and EEG measurement of sleepiness. *Int. J. Psychophysiol.* 52 97–112. 10.1016/j.ijpsycho.2003.12.007 15003376

[B99] Moon P. Spencer D. E. (1944). On the stiles-crawford effect. *J. Opt. Soc. Am.* 34:319. 10.1364/JOSA.34.000319

[B100] Morad Y. Lemberg H. Yofe N. Dagan Y. (2000). Pupillography as an objective indicator of fatigue. *Curr. Eye Res.* 21 535–542. 10.1076/0271-3683(200007)2111-ZFT535 11035533

[B101] Morimoto C. H. Amir A. Flickner M. (2002). “Detecting eye position and gaze from a single camera and 2 light sources,” in *Proceedings of the International Conference on Pattern Recognition*, Quebec City, QC, 314–317. 10.1109/icpr.2002.1047459

[B102] Morimoto C. H. Koons D. Amir A. Flickner M. (2000). Pupil detection and tracking using multiple light sources. *Image Vis. Comput.* 18 331–335. 10.1016/S0262-8856(99)00053-0

[B103] Münch M. Léon L. Crippa S. V. Kawasaki A. (2012). Circadian and wake-dependent effects on the pupil light reflex in response to narrow-bandwidth light pulses. *Investig. Ophthalmol. Vis. Sci.* 53 4546–4555. 10.1167/iovs.12-9494 22669721

[B104] Mure L. S. (2021). Intrinsically photosensitive retinal ganglion cells of the human retina. *Front. Neurol.* 12: 636330. 10.3389/fneur.2021.636330 33841306 PMC8027232

[B105] Murphy P. R. Vandekerckhove J. Nieuwenhuis S. (2014). Pupil-linked arousal determines variability in perceptual decision making. *PLoS Comput. Biol.* 10: e1003854. 10.1371/journal.pcbi.1003854 25232732 PMC4168983

[B106] Murray I. J. Kremers J. McKeefry D. Parry N. R. A. (2018). Paradoxical pupil responses to isolated M-cone increments. *J. Opt. Soc. Am. A* 35:B66. 10.1364/josaa.35.000b66 29603924

[B107] Murray N. P. Hunfalvay M. Bolte T. (2017). The reliability, validity, and normative data of interpupillary distance and pupil diameter using eye-tracking technology. *Transl. Vis. Sci. Technol.* 6:2. 10.1167/tvst.6.4.2 28685104 PMC5497600

[B108] OpenCV (2020). *OpenCV: Camera Calibration and 3D Reconstruction.* Available online at: https://docs.opencv.org/master/d9/d0c/group__calib3d.html (accessed November 16, 2020).

[B109] Ostrin L. A. Abbott K. S. Queener H. M. (2017). Attenuation of short wavelengths alters sleep and the ipRGC pupil response. *Ophthalmic Physiol. Opt.* 37 440–450. 10.1111/opo.12385 28656675 PMC7229994

[B110] Pedrotti M. Lei S. Dzaack J. Rötting M. (2011). A data-driven algorithm for offline pupil signal preprocessing and eyeblink detection in low-speed eye-tracking protocols. *Behav. Res. Methods* 43 372–383. 10.3758/s13428-010-0055-7 21302023

[B111] Pedrotti M. Mirzaei M. A. Tedesco A. Chardonnet J. R. Mérienne F. Benedetto S. (2014). Automatic stress classification with pupil diameter analysis. *Int. J. Hum. Comput. Interact.* 30 220–236. 10.1080/10447318.2013.848320

[B112] Pérez A. Córdoba M. L. García A. Méndez R. Muñoz M. L. Pedraza J. L. (2003). *A Precise Eye-Gaze Detection and Tracking System.* Plzen, Czech Republic.

[B113] Pinheiro H. M. da Costa R. M. (2021). Pupillary light reflex as a diagnostic aid from computational viewpoint: a systematic literature review. *J. Biomed. Inform.* 117:103757. 10.1016/j.jbi.2021.103757 33826949

[B114] Provencio I. Jiang G. De Grip W. J. Pär Hayes W. Rollag M. D. (1998). Melanopsin: an opsin in melanophores, brain, and eye. *Proc. Natl. Acad. Sci. U.S.A.* 95 340–345. 10.1073/pnas.95.1.340 9419377 PMC18217

[B115] Provencio I. Rodriguez I. R. Jiang G. Hayes W. P. Moreira E. F. Rollag M. D. (2000). A novel human opsin in the inner retina. *J. Neurosci.* 20 600–605. 10.1523/JNEUROSCI.20-02-00600.2000 10632589 PMC6772411

[B116] Rao F. Chan A. H. S. Zhu X. F. (2017). Effects of photopic and cirtopic illumination on steady state pupil sizes. *Vision Res.* 137 24–28. 10.1016/j.visres.2017.02.010 28688906

[B117] Rea M. S. Figueiro M. G. (2018). Light as a circadian stimulus for architectural lighting. *Light. Res. Technol.* 50 497–510. 10.1177/1477153516682368

[B118] Reeves P. (1918). Rate of pupillary dilation and contraction. *Psychol. Rev.* 25 330–340. 10.1037/h0075293

[B119] Rote G. (1991). Computing the minimum Hausdorff distance between two point sets on a line under translation. *Inf. Process. Lett.* 38 123–127. 10.1016/0020-0190(91)90233-8

[B120] Ruby N. F. Brennan T. J. Xie X. Cao V. Franken P. Heller H. C. (2002). Role of melanopsin in circadian responses to light. *Science* 298 2211–2213. 10.1126/science.1076701 12481140

[B121] Rukmini A. V. Milea D. Aung T. Gooley J. J. (2017). Pupillary responses to short-wavelength light are preserved in aging. *Sci. Rep.* 7 1–9. 10.1038/srep43832 28266650 PMC5339857

[B122] Sagawa K. (2006). Toward a CIE supplementary system of photometry: brightness at any level including mesopic vision. *Ophthalmic Physiol. Opt.* 26 240–245. 10.1111/j.1475-1313.2006.00357.x 16684150

[B123] San Agustin J. Skovsgaard H. Mollenbach E. Barret M. Tall M. Hansen D. W. (2010). “Evaluation of a low-cost open-source gaze tracker,” in *Proceedings of the 2010 Symposium on Eye-Tracking Research & Applications*, Austin, TX, 77–80. 10.1145/1743666.1743685

[B124] Santini T. Fuhl W. Geisler D. Kasneci E. (2017). “EyeRecToo: open-source software for real-time pervasive head-mounted eye tracking,” in *VISIGRAPP 2017 Proceedings of the 12th International Joint Conference on Computer Vision, Imaging and Computer Graphics Theory and Applications*, (Setúbal: SciTePress), 96–101. 10.5220/0006224700960101

[B125] Santini T. Fuhl W. Kasneci E. (2018a). PuRe: robust pupil detection for real-time pervasive eye tracking. *Comput. Vis. Image Underst.* 170 40–50. 10.1016/j.cviu.2018.02.002

[B126] Santini T. Fuhl W. Kasneci E. (2018b). “PuReST: robust pupil tracking for real-time pervasive eye tracking,” in *Proceedings of the 2018 ACM Symposium on Eye Tracking Research & Applications (ETRA), 2018*, Warsaw. 10.1145/3204493.3204578

[B127] Schluroff M. Zimmermann T. E. Freeman R. B. Hofmeister K. Lorscheid T. Weber A. (1986). Pupillary responses to syntactic ambiguity of sentences. *Brain Lang.* 27 322–344. 10.1016/0093-934X(86)90023-4 3513899

[B128] Schmidt T. M. Alam N. M. Chen S. Kofuji P. Li W. Prusky G. T. (2014). A role for melanopsin in alpha retinal ganglion cells and contrast detection. *Neuron* 82 781–788. 10.1016/j.neuron.2014.03.022 24853938 PMC4083763

[B129] Schneider M. Elbau I. G. Nantawisarakul T. Pöhlchen D. Brückl T. BeCOME Working (2020). Pupil dilation during reward anticipation is correlated to depressive symptom load in patients with major depressive disorder. *Brain Sci.* 10:906. 10.3390/brainsci10120906 33255604 PMC7760331

[B130] Schwalm M. Jubal E. R. (2017). Back to pupillometry: how cortical network state fluctuations tracked by pupil dynamics could explain neural signal variability in human cognitive neuroscience. *eNeuro* 4: ENEURO.0293-16.2017. 10.1523/ENEURO.0293-16.2017 29379876 PMC5788057

[B131] Schwarz L. Gamba H. R. Pacheco F. C. Ramos R. B. Sovierzoski M. A. (2012). “Pupil and iris detection in dynamic pupillometry using the OpenCV library,” in *Proceedings of the 2012 5th Int. Congr. Image Signal Process. CISP 2012*, Chongqing, 211–215. 10.1109/CISP.2012.6469846

[B132] Schwiegerling J. (2000). Theoretical limits to visual performance. *Surv. Ophthalmol.* 45 139–146. 10.1016/S0039-6257(00)00145-4 11033040

[B133] Sharpe L. T. Stockman A. Jagla W. Jaägle H. (2005). A luminous efficiency function, V^∗^(λ), for daylight adaptation. *J. Vis.* 5 948–968. 10.1167/5.11.3 16441195

[B134] Sibley C. Foroughi C. K. Brown N. L. Phillips H. Drollinger S. Eagle M. (2020). More than means: characterizing individual differences in pupillary dilations. *Proc. Hum. Factors Ergon. Soc. Annu. Meet.* 64 57–61. 10.1177/1071181320641017

[B135] Smith V. C. Pokorny J. Lee B. B. Dacey D. M. (2008). Sequential processing in vision: the interaction of sensitivity regulation and temporal dynamics. *Vision Res.* 48 2649–2656. 10.1016/j.visres.2008.05.002 18558416 PMC2627776

[B136] Solomon S. G. Lennie P. (2007). The machinery of colour vision. *Nat. Rev. Neurosci.* 8 276–286. 10.1038/nrn2094 17375040

[B137] Spitschan M. (2019a). Melanopsin contributions to non-visual and visual function. *Curr. Opin. Behav. Sci.* 30 67–72. 10.1016/j.cobeha.2019.06.004 31396546 PMC6687502

[B138] Spitschan M. (2019b). Photoreceptor inputs to pupil control. *J. Vis.* 19:5. 10.1167/19.9.5 PMC6699792 31415056

[B139] Spitschan M. Lazar R. Yetik E. Cajochen C. (2019). No evidence for an S cone contribution to acute neuroendocrine and alerting responses to light. *Curr. Biol.* 29 R1297–R1298. 10.1016/j.cub.2019.11.031 31846672 PMC6926470

[B140] Stanley P. Davies A. (1995). The effect of field of view size on steady-state pupil diameter. *Ophthalmic Physiol. Opt.* 15 601–603. 10.1016/0275-5408(94)00019-V 8594531

[B141] Stockman A. Sharpe L. T. (2000). The spectral sensitivities of the middle- and long-wavelength-sensitive cones derived from measurements in observers of known genotype. *Vision Res.* 40 1711–1737. 10.1016/S0042-6989(00)00021-3 10814758

[B142] Świrski L. Bulling A. Dodgson N. (2012). “Robust real-time pupil tracking in highly off-axis images,” in *ETRA ’12: Proceedings of the Symposium on Eye Tracking Research and Applications*, Santa Barbara, CA, 173–176. 10.1145/2168556.2168585

[B143] Świrski L. Dodgson N. A. (2013). “A fully-automatic, temporal approach to single camera, glint-free 3D eye model fitting,” in *Proceedings of the Pervasive Eye Track. Mob. Eye-Based Interact*, Lund.

[B144] Tabashum T. Zaffer A. Yousefzai R. Colletta K. Jost M. B. Park Y. (2021). Detection of Parkinson’s disease through automated pupil tracking of the post-illumination pupillary response. *Front. Med.* 8:645293. 10.3389/fmed.2021.645293 33842509 PMC8026862

[B145] Tähkämö L. Partonen T. Pesonen A. K. (2019). Systematic review of light exposure impact on human circadian rhythm. *Chronobiol. Int.* 36 151–170. 10.1080/07420528.2018.1527773 30311830

[B146] Thaler L. Schütz A. C. Goodale M. A. Gegenfurtner K. R. (2013). What is the best fixation target? The effect of target shape on stability of fixational eye movements. *Vision Res.* 76 31–42. 10.1016/j.visres.2012.10.012 23099046

[B147] Thapan K. Arendt J. Skene D. J. (2001). An action spectrum for melatonin suppression: evidence for a novel non-rod, non-cone photoreceptor system in humans. *J. Physiol.* 535 261–267. 10.1111/j.1469-7793.2001.t01-1-00261.x 11507175 PMC2278766

[B148] Titz J. Scholz A. Sedlmeier P. (2018). Comparing eye trackers by correlating their eye-metric data. *Behav. Res. Methods* 50 1853–1863. 10.3758/s13428-017-0954-y 28879442

[B149] Tkacz-Domb S. Yeshurun Y. (2018). The size of the attentional window when measured by the pupillary response to light. *Sci. Rep.* 8 1–7. 10.1038/s41598-018-30343-7 30089801 PMC6082875

[B150] Topal C. Cakir H. I. Akinlar C. (2017). *APPD.* Available online at: http://arxiv.org/abs/1709.06366 (accessed 28th December, 2020).

[B151] Truong W. Zandi B. Trinh V. Q. Khanh T. Q. (2020). Circadian metric – Computation of circadian stimulus using illuminance, correlated colour temperature and colour rendering index. *Build. Environ.* 184:107146. 10.1016/j.buildenv.2020.107146

[B152] Tsukahara J. S. Harrison T. L. Engle R. W. (2016). The relationship between baseline pupil size and intelligence. *Cogn. Psychol.* 91 109–123. 10.1016/j.cogpsych.2016.10.001 27821254

[B153] Van der Stoep N. Van der Smagt M. J. Notaro C. Spock Z. Naber M. (2021). The additive nature of the human multisensory evoked pupil response. *Sci. Rep.* 11:707. 10.1038/s41598-020-80286-1 33436889 PMC7803952

[B154] Van Egroo M. Gaggioni G. Cespedes-Ortiz C. Ly J. Q. M. Vandewalle G. (2019). Steady-state pupil size varies with circadian phase and sleep homeostasis in healthy young men. *Clocks Sleep* 1 240–258. 10.3390/clockssleep1020021 33089167 PMC7445830

[B155] Van Meeteren A. (1978). On the detective quantum efficiency of the human eye. *Vision Res.* 18 257–267. 10.1016/0042-6989(78)90160-8 664301

[B156] van Rij J. Hendriks P. van Rijn H. Baayen R. H. Wood S. N. (2019). Analyzing the time course of pupillometric data. *Trends Hear.* 23 1–22. 10.1177/2331216519832483 31081486 PMC6535748

[B157] Vera-Olmos F. J. Pardo E. Melero H. Malpica N. (2018). DeepEye: deep convolutional network for pupil detection in real environments. *Integr. Comput. Aided. Eng.* 26 85–95. 10.3233/ICA-180584

[B158] Wang D. Mulvey F. B. Pelz J. B. Holmqvist K. (2017). A study of artificial eyes for the measurement of precision in eye-trackers. *Behav. Res. Methods* 49 947–959. 10.3758/s13428-016-0755-8 27383751

[B159] Watson A. B. Yellott J. I. (2012). A unified formula for light-adapted pupil size. *J. Vis.* 12 1–16. 10.1167/12.10.12 23012448

[B160] Wildemeersch D. Baeten M. Peeters N. Saldien V. Vercauteren M. Hans G. (2018). Pupillary dilation reflex and pupillary pain index evaluation during general anaesthesia: a pilot study. *Rom. J. Anaesth. Intensive Care* 25 19–23. 10.21454/rjaic.7518.251.wil 29756058 PMC5931178

[B161] Winn M. B. Wendt D. Koelewijn T. Kuchinsky S. E. (2018). Best practices and advice for using pupillometry to measure listening effort: an introduction for those who want to get started. *Trends Hear.* 22:233121651880086. 10.1177/2331216518800869 30261825 PMC6166306

[B162] Withouck M. Smet K. A. G. Ryckaert W. R. Pointer M. R. Deconinck G. Koenderink J. (2013). Brightness perception of unrelated self-luminous colors. *J. Opt. Soc. Am. A* 30:1248. 10.1364/JOSAA.30.001248 24323112

[B163] Woodhouse J. M. (1975). The effect of pupil size on grating detection at various contrast levels. *Vision Res.* 15 645–648. 10.1016/0042-6989(75)90278-3 1138478

[B164] Yiu Y. H. Aboulatta M. Raiser T. Ophey L. Flanagin V. L. Eulenburg P. (2019). DeepVOG: open-source pupil segmentation and gaze estimation in neuroscience using deep learning. *J. Neurosci. Methods* 324:108307. 10.1016/j.jneumeth.2019.05.016 31176683

[B165] Young R. S. L. Kimura E. (2008). Pupillary correlates of light-evoked melanopsin activity in humans. *Vision Res.* 48 862–871. 10.1016/j.visres.2007.12.016 18262584

[B166] Zandi B. Eissfeldt A. Herzog A. Khanh T. Q. (2021). Melanopic limits of metamer spectral optimisation in multi-channel smart lighting systems. *Energies* 14:527. 10.3390/en14030527

[B167] Zandi B. Guo X. Bodrogi P. Khanh T. Q. (2018). “EXPERIMENTAL EVALUATION OF DIFFERENT BRIGHTNESS PERCEPTION MODELS BASED ON HUMAN PUPIL LIGHT RESPONSES,” in *PROCEEDINGS OF CIE 2018 TOPICAL CONFERENCE ON SMART LIGHTING*, (Taipei: International Commission on Illumination, CIE), 201–208. 10.25039/x45.2018.OP34

[B168] Zandi B. Khanh T. Q. (2021). Deep learning-based pupil model predicts time and spectral dependent light responses. *Sci. Rep.* 11:841. 10.1038/s41598-020-79908-5 33436693 PMC7803766

[B169] Zandi B. Klabes J. Khanh T. Q. (2020). Prediction accuracy of L- and M-cone based human pupil light models. *Sci. Rep.* 10:10988. 10.1038/s41598-020-67593-3 32620793 PMC7335057

[B170] Zele A. J. Adhikari P. Cao D. Feigl B. (2019). Melanopsin and cone photoreceptor inputs to the afferent pupil light response. *Front. Neurol.* 10:529. 10.3389/fneur.2019.00529 31191431 PMC6540681

[B171] Zhang Z. (2000). A flexible new technique for camera calibration. *IEEE Trans. Pattern Anal. Mach. Intell.* 22 1330–1334. 10.1109/34.888718

[B172] Zhu D. Moore S. T. Raphan T. (1999). Robust pupil center detection using a curvature algorithm. *Comput. Methods Programs Biomed.* 59 145–157. 10.1016/S0169-2607(98)00105-9 10386764

