# Supplementary material for: PupilEXT: Flexible Open-Source Platform for High-Resolution Pupillometry in Vision Research
Source: Front Neurosci. 2021 Jun 18;15:676220. doi: 10.3389/fnins.2021.676220 (PMC8249868; doi:10.3389/fnins.2021.676220)
Supplement: Supplementary file 1 [file Table_1.DOCX]

Supplementary Material

Three videos are provided to illustrate the handling and the features of the *PupilEXT* software. The first video introduces the *PupilEXT* software in stereo vision mode. The second video shows how the pupillometry can be carried out by using a single camera. The third video shows how pupillometry can be carried out with existing eye images without connected cameras. All videos and tutorials on how to set up the pupillometry platform are available at the GitHub repository of *PupilEXT*: <https://github.com/openPupil/Open-PupilEXT>
